# The broad assessment of HCV genotypes 1 and 3 antigenic targets reveals limited cross-reactivity with implications for vaccine design

**DOI:** 10.1136/gutjnl-2014-308724

**Published:** 2015-06-19

**Authors:** Annette von Delft, Isla S Humphreys, Anthony Brown, Katja Pfafferott, Michaela Lucas, Paul Klenerman, Georg M Lauer, Andrea L Cox, Silvana Gaudieri, Eleanor Barnes

**Affiliations:** 1 NDM, University of Oxford, Oxford, Oxfordshire, UK; 2 Institute of Immunology and Infectious Diseases, Murdoch University, Perth, Western Australia, Australia; 3 School of Medicine and Pharmacology, Harry Perkins Institute, University of Western Australia, Western Australia, Australia; 4 School of Pathology and Laboratory Medicine, University of Western Australia, Western Australia, Australia; 5 Ragon Institute of MGH, Boston, Massachusetts, USA; 6 John Hopkins University, Baltimore, Maryland, USA; 7 School of Anatomy, Physiology and Human Biology, University of Western Australia, Perth, Western Australia, Australia

**Keywords:** HCV, GENOTYPE, CELLULAR IMMUNITY

## Abstract

**Objective:**

Developing a vaccine that is cross-reactive between HCV genotypes requires data on T cell antigenic targets that extends beyond genotype-1. We characterised T cell immune responses against HCV genotype-3, the most common infecting genotype in the UK and Asia, and assessed within genotype and between genotype cross-reactivity.

**Design:**

T cell targets were identified in 140 subjects with either acute, chronic or spontaneously resolved HCV genotype-3 infection using (1) overlapping peptides and (2) putative human leucocyte antigens (HLA)-class-I wild type and variant epitopes through the prior assessment of polymorphic HCV genomic sites associated with host HLA, in IFNγ-ELISpot assays. CD4+/CD8+ T cell subsets were defined and viral variability at T cell targets was determined through population analysis and viral sequencing. T cell cross-reactivity between genotype-1 and genotype-3 variants was assessed.

**Results:**

In resolved genotype-3 infection, T cells preferentially targeted non-structural proteins at a high magnitude, whereas in chronic disease T cells were absent or skewed to target structural proteins. Additional responses to wild type but not variant HLA predicted peptides were defined. Major sequence viral variability was observed within genotype-3 and between genotypes 1 and 3 HCV at T cell targets in resolved infection and at dominant epitopes, with limited T cell cross-reactivity between viral variants. Overall 41 CD4/CD8+ genotype-3 T cell targets were identified with minimal overlap with those described for HCV genotype-1.

**Conclusions:**

HCV T cell specificity is distinct between genotypes with limited T cell cross-reactivity in resolved and chronic disease. Therefore, viral regions targeted in natural HCV infection may not serve as attractive targets for a vaccine that aims to protect against multiple HCV genotypes.

**Significance of this study**

**What is already known on this subject?**

- HCV genotype-3 is the most prevalent HCV strain in South Asia and the UK.
- HCV viral genotypes share approximately 80% sequence homology.
- Limited data on T cell specificity is available for HCV genotypes other than HCV genotype-1.
- Population studies assessing the association of HLA-class-I with viral genomic polymorphisms suggest that T cell specificity differs between genotypes 1 and 3.

**What are the new findings?**

- A comprehensive assessment of HCV genotype-3 T cell specificity identifies 41 CD4+ and CD8+ genotype-3 specific T cell targets across the viral genome.
- A novel sequence led approach can be used to identify HLA-class-I epitopes under T cell selection.
- T cell targets in HCV genotype-3 infection are distinct from those targeted in HCV genotype-1 in resolved and chronic disease.
- T cell cross-reactivity to genotype-1 and genotype-3 sequence variants in resolved infection and at dominant HCV genotype-3 epitopes is limited.

**How might it impact on clinical practice in the future?**

- Distinct T cell specificity and limited T cell cross-reactivity between HCV genotypes are important considerations for the development of vaccines aiming to induce T cell responses cross-reactive against multiple HCV genotypes.

## Introduction

HCV infection is a major health risk, infecting approximately 170 million people worldwide.^1^ The majority of infected patients develop persistent infection, which may lead to liver cirrhosis, hepatocellular cancer and death.^2^ Even though major advances in HCV treatment with directly acting antivirals (DAAs) have been achieved over recent years,^3^ ^4^ ^5^ costs are high and treatment may remain inaccessible for many, particularly in health resource poor countries. In addition, reinfection with DAA resistant variants can occur following successful therapy.^6^ An effective prophylactic HCV vaccine remains an unmet clinical need.

HCV is a highly genetically diverse pathogen that is divided into 7 major genotypes and 67 subtypes^7^ that are broadly distributed by geographical location. Even within a single host infected with one subtype HCV exists as multiple closely related but distinct viral quasi species.^8^ HCV genotype-1 is the dominant genotype globally,^9^ but HCV genotype-3 is now the major infecting genotype in the UK^10^ and in large parts of Asia,^1^ infecting approximately 53 million people globally^9^ and commonly associated with injecting drug use and interventional medical practice.^11–13^

HCV genotype-1 vaccines based on viral vectored technology used in heterologous prime/boost regimens are currently in development and able to induce a high magnitude of T cells that target multiple HCV antigens.^14^ ^15^ However, the success of T cell vaccines in regions where multiple HCV genotypes coexist will depend on the generation of T cells that have the capacity to target multiple viral strains or viral regions that are conserved between genotypes. A better understanding of genotype-specific immune responses will therefore aid the development of vaccines active against multiple genotypes.

Based on significant viral genetic differences between HCV genotypes,^7^ we hypothesised that T cell targets differ in HCV genotypes 1 and 3. Comparative studies on HCV genotype-1 and HCV genotype-3-specific T cell immunity to date include an analysis of genotype-specific sequence polymorphisms linked to HLA types, suggesting that T cell targets are distinct between HCV genotypes.^16^ However T cell immune pressure was not confirmed by experimental T cell assays. Although patients infected with HCV genotype-3a have been included in numerous publications addressing HCV specific T cell immunity experimentally, studies using specific HCV genotype-3 cohorts and genotype-3 peptide sets are limited to a single study evaluating T cell responses to the NS3 protein only^17^ and our previous study that primarily evaluated the impact of therapy on HCV genotype-3-specific T cell responses.^18^

To date, no specific cross-reactive T cell targets linked to spontaneous resolution of infection have been described, and no comprehensive assessment of T cell cross-reactivity between HCV genotypes 1 and 3 has been performed. Even if T cell targets are shared between genotypes, a single amino acid (AA) substitution may abort or substantially decrease recognition of the epitope by wild type primed T cells.^19–21^ Although some T cell cross-reactivity between HCV genotypes has been described,^17^ ^18^ several small-scale studies in patients with evidence of multiple infections have shown lack of cross-reactivity of CD4+ T cell responses between genotypes.^22^ ^23^ Furthermore, systematic analysis assessing all possible sequence variants between genotypes is limited to a singe epitope, showing that CD8+ T cells primed against the dominant HCV genotype-1 epitope NS3_1073_ do not recognise HCV genotype-2 and genotype-3 viral variants at that location.^21^

T cell cross-reactivity between heterologous viral strains can also be evaluated in the context of human reinfection observational studies and chimpanzee rechallenge experiments. Published studies suggest that chimpanzees^24^ ^25^ and humans^26^ that spontaneously clear acute HCV infection are more likely to clear subsequent infections. However the role of cross-reactivity in preventing chronic disease upon reinfection is not clear and while clearance of heterologous HCV reinfection is reported,^24^ persistent infection on rechallenge with heterologous strains is also common.^25^ ^27^ Furthermore, these studies have not evaluated T cell cross-reactivity at epitope level, and other factors may explain the phenomenon of repeated viral resolution such as a favourable innate immune response and host genetic make up.^24^

ELISpot assays have been established as a reliable method to define T cell epitopes in chronic viral infections^18^ ^28^ and T cell vaccine studies.^15^ ^29^ Overlapping peptides homologous with the pathogen genome are commonly used as a screening tool to identify T cell epitopes. However, it has been reported previously that the detection of T cell responses in IFNγ-ELISpot assays may be dependent on the position of the presented optimal epitope within an overlapping peptide^30^ and T cell responses may be missed when screening with this approach. To address this, we assessed T cell responses using two complementary HCV genotype-3-specific peptide sets; one based on a novel, sequence-led approach using wild type and variant peptides corresponding to putative HLA class-I restricted epitopes under T cell selection identified in a large HCV genotype-3 sequence data set;^16^ the other based on a consensus sequence derived from 15 chronically genotype-3 infected patients spanning the whole HCV genome.^18^

We aimed to comprehensively characterise T cell immune responses against HCV genotype-3 and to compare T cell specificity between HCV genotype-1 and genotype-3. Finally we assessed T cell cross-reactivity between common HCV genotype-1 and genotype-3 sequence variants, focusing particularly on dominant genotype-3 T cell epitopes, in addition to a cohort of patients with resolved infection where cross-reactive T cells associated with viral control may have the greatest implications for vaccine design.

## Methods

### Patient cohort

One hundred and forty HCV genotype-3a infected individuals including 16 acutely infected, 108 chronically infected and 16 with spontaneously resolved infection were recruited (John Radcliffe Hospital Oxford, MGH Boston, and the BBAASH cohort, Baltimore^31^). Informed consent and local ethical approval was obtained for all patients. Patient details are summarised in online supplementary table S1. Acute patients were defined as those within the first 6 months of infection (n=16), of whom 12 were not treated (n=4 cleared infection spontaneously; n=6 proceeded to chronic infection; n=2 lost to follow-up), and 4 were treated during acute infection (n=3 sustained virological response; n=1 non-responder) (see online supplementary table S2). HCV genotype could not be determined in spontaneously resolved individuals by conventional genotyping; however, to define the infecting genotype, T cell responses to genotype-1 and genotype-3 peptides were assessed in this group.

### Peptide sets and approaches used to identify HCV-specific T cell targets

(1) *Overlapping peptides* for HCV genotype-1 and genotype-3: A genotype-1b peptide set containing peptides 15 to 18 AA in length overlapping by 10 AA derived from HCV J4 sequence (AF054250); A genotype-3a peptide set based on 18 full-length genotype-3a sequences as previously described, spanning the whole viral genome (GQ356200-GQ356215, GQ356217 and JF509175-JF509177).^18^

(2) *HLA-predicted peptides* for genotype-3 were based on a novel sequence led peptide design approach aiming to identify HLA class-I restricted optimal epitopes: Associations between HLA-class-I alleles and HCV viral sequence polymorphisms within NS2-NS5B were identified in a cohort of 136 HCV genotype-3a infected patients.^16^ Epitope computer prediction programmes were used to identify putative T cell epitopes (9–10AA) hosting HLA-associated polymorphic sites (BioInformatics and Molecular Analysis Section (BIMAS) score ≥50, Syfpeithi score ≥20; http://www-bimas.cit.nih.gov, http://www.syfpeithi.de). Fifty-five epitopes were predicted to contain HLA-associated polymorphic sites within the peptide (see online supplementary table S3), whereas in 10 peptides the polymorphic site was flanking the epitope (see online supplementary table S4). Wild type (defined as the consensus AA at each position in an HCV genotype-3 sequence alignment)^16^ and variant (defined as the second most common AA at each position linked to patient HLA) peptides were subsequently evaluated in T cell assays matched to the patients’ HLA type. We have previously published T cell responses in 10 spontaneously resolved and 17 chronically HCV genotype-3 infected patients using overlapping peptides only^18^; these have been included in this manuscript for assessment using HLA-predicted peptides and comparative analysis of T cell specificity.

Detected T cell responses to overlapping peptide pools (HCV core, E1, E2, p7/NS2, NS3 protease (NS3p), NS3 helicase (NS3h), NS4, proximal NS5B (NS5BI), and distal NS5B (NS5BII)) were mapped to subpools and single peptides. T cell responses to both peptide sets were compared at pool level, and at single epitope level in patients with mapped responses. CD4+/CD8+ restriction was defined using CD8+ depletion assays and intracellular staining assays as previously described.^18^ For further analyses, dominant responses were defined as those targeted in more than four patients within the Oxford cohort.

### HLA typing

DNA was extracted using the DNeasy Blood And Tissue Kit (Qiagen) from peripheral blood mononuclear cells (PBMCs) or whole blood using the Gentra Puregene kit (Qiagen) as per manufacturer’s instructions and then HLA typed (Transplant Immunology Lab, Oxford Radcliffe Hospitals).^32^

### ELISpot assays

Human PBMCs were separated, frozen immediately and stored in liquid nitrogen as previously described.^18^ T cell responses were assessed using thawed PBMCs in IFNγ-ELISpot assays as previously described.^33^ In brief, precoated ELISpot plates (anti-IFNγ monoclonal antibody (0.5 μg/well, Mabtech)) were blocked with R10 (RPMI Sigma, 10% fetal calf serum (FCS), penicillin and streptomycin added). For 18 h, 200 000 PBMCs/well were stimulated with HCV genotype-3 peptide sets (3 μg/mL), cytomegalovirus (CMV) lysate (0.05 μg/mL, Chiron), influenza, Epstein Barr virus and CMV (FEC) CD8+ epitopes in a single pool (3 μg/mL BEI resources) in duplicates for each condition. Dimethyl sulfoxide (DMSO) and concanavalin A (10 μg, Sigma) served as negative and positive controls, respectively; all ELISpot assays were strongly positive for concanavalin A. Additionally, 101/140 patients were positive for CMV lysate and 68/140 patients were positive for FEC antigens (mean spot-forming units/10^6^PBMCs 661.77 and 686.45). All patients were tested using overlapping peptide pools. HLA-predicted peptides were tested in HLA-typed patients with cells available. SFUs were counted on an automated ELISpot plate reader. A positive cut-off of 40 SFUs/10^6^PBMCs for the HCV genotype-3 peptides and 43 SFUs/10^6^PBMCs for genotype-1b peptides was defined previously in healthy volunteers using; (mean SFU/10^6^PBMCs in test wells—negative control wells)+3×SD.^18^

### Viral sequencing

HCV viral sequencing was performed as previously published.^18^ In brief, patient plasma was concentrated by centrifugation (1 h, 23 000 rpm, 4°C) and viral RNA was extracted using a QIAmp Viral RNA Mini Kit (Qiagen). Reverse-transcription and first round PCR were performed in a single step (Superscript III OneStep RT-PCR system, Platinum Taq enzyme (Invitrogen)). In a second step single proteins were amplified in multiple nested PCR reactions (High Fidelity Taq DNA polymerase (Roche), for primers see refs. 18 and 34). Amplified PCR fragments were gel purified and sequenced bidirectionally with Prism Big Dye (Applied Biosystems) on an ABI3100 DNA automated sequencer. Sequences were edited using the Sequencher 4.8 Software (Gene Codes), and aligned using Se-Al (http://tree.bio.ed.ac.uk). Sequence entropy was calculated using the Shannon entropy score (http://evolve.zoo.ox.ac.uk/Evolve/SHiAT.html) using HCV genotype-3 sequences derived from the Los Alamos sequence database (http://hcv.lanl.gov/content/index).35

### Analysis of HCV genotype-1 and genotype-3 T cell targets

HCV genotype-1 and genotype-3 epitopes were obtained from the immune epitope database resource (IEDB, http://www.iedb.com). To ensure data quality, epitopes were crosschecked with primary publications; epitope duplications, sequence variants and epitopes described in non-human organisms were excluded. Dominant HCV genotype-1 targets were defined as those described in more than five publications, and were compared with all genotype-3 epitopes defined in this study. Experimentally identified HCV genotype-3 targets were compared with all HCV genotype-1 epitopes described on the IEDB. Targets previously described were defined as ‘overlapping’ with those detected experimentally if epitopes exhibited >80% AA sequence homology or ‘not overlapping’ if <80% AAs sequence homology, or if epitopes overlapped by less than 7 AA (left coloured bar, online supplementary tables S7 and S8).

### Statistical analysis

Non-parametrical tests were used throughout, paired for within-individual comparisons (Wilcoxon) and unpaired for group comparisons (Mann-Whitney). A p value of <0.05 was considered statistically significant. Prism (V.4.0 for Mac) was used.

## Results

### T cell specificity differs in patients with spontaneously resolved and chronic HCV genotype-3 infection

**Figure 1:**
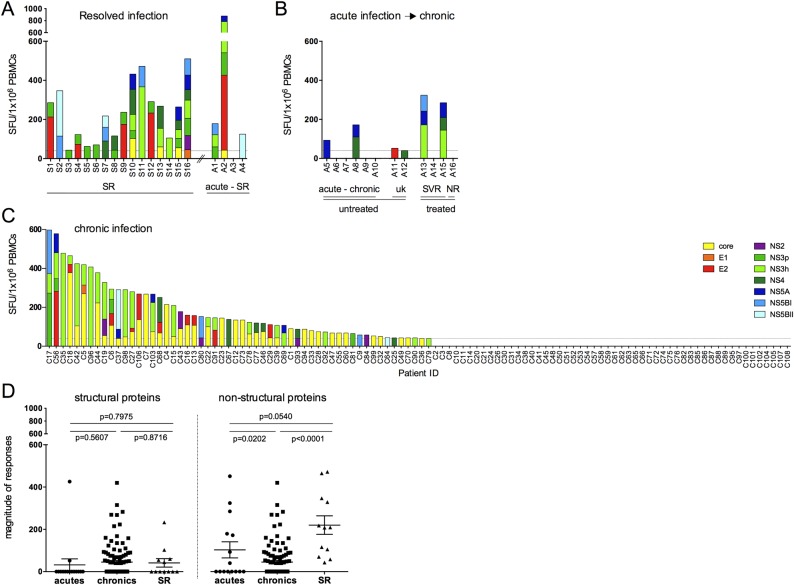
T cell responses against an HCV genotype-3 overlapping peptide set in HCV genotype-3 infected patients. HCV genotype-3 specific T cell responses were measured by IFNγ-ELISpot assays (SFU/10^6^ PBMCs) using an HCV genotype-3-specific peptide set spanning the entire HCV genome. T cell responses over cut-off were detected in (A) 16/16 individuals with spontaneously resolved infection and 3/4 patients with acute infection that subsequently spontaneously resolved infection (acutely infected→SR); (B) 6/12 patients with acute HCV genotype-3 infection that did not clear infection; and (C) 56/108 individuals chronically infected with HCV genotype-3. (D) Comparison of total magnitude of T cell response in IFNγ-ELISpot assays to HCV structural and non-structural viral regions is depicted. SFU, spot forming units; NS3p NS3 protease; NS3h NS3 helicase; NS5BI proximal NS5B region; NS5BII distal NS5B region; ns, not significant; PBMC, peripheral blood mononuclear cell; SR, spontaneously resolved patients; C, chronic; uk, unknown; SVR, sustained virological response; NR, non-responder. P values are given between patient groups.

T cell responses to HCV genotype-3 were first assessed in 20 patients with spontaneously resolved infection since these responses are most likely to be causally related to viral resolution. We included four patients with acute infection, assessed at the earliest available time point after presentation, who subsequently resolved infection. Using HCV genotype-3 peptides, T cell responses were identified in 19/20 (95%) patients targeting a broad range of viral regions (figure 1A), as reported for spontaneously resolved HCV genotype-1 infection.^35^ ^36^ In patients with acute HCV genotype-3 infection who did not resolve infection, T cell responses were identified in 6/12 patients (50%), predominantly targeting non-structural proteins (figure 1B). In chronic HCV genotype-3 infection T cell responses were detected in 56/108 patients that mainly targeted the HCV core (39/56) and NS3 proteins (26/56). Similar to previous data in HCV genotype-1, no T cell responses were detected in 48% of HCV genotype-3 chronically infected individuals (figure 1C).^18^ ^37^ As previously described in HCV genotype-1, the total magnitude of T cell responses in spontaneously resolved infection was significantly stronger and targeted more viral peptide pools compared with chronic infection (p<0.0001, online supplementary figure S1A).^35^ In defining T cell specificity, we observed that patients with resolved infection preferentially targeted HCV non-structural regions and at higher magnitude compared with patients with chronic infection, whereas the magnitude of responses to HCV structural proteins did not differ between patient groups (see figure 1D and online supplementary figure S1B).

### Additional HCV genotype-3 T cell targets are identified using putative HLA class-I peptides associated with viral genomic polymorphisms

Using overlapping peptides, T cell responses were mapped to individual peptides in 55 patients (see online supplementary tables S5 and S6); 35 HCV genotype-3-specific T cell targets were identified, 10 located in HCV structural and 25 in non-structural regions.

**Figure 2:**
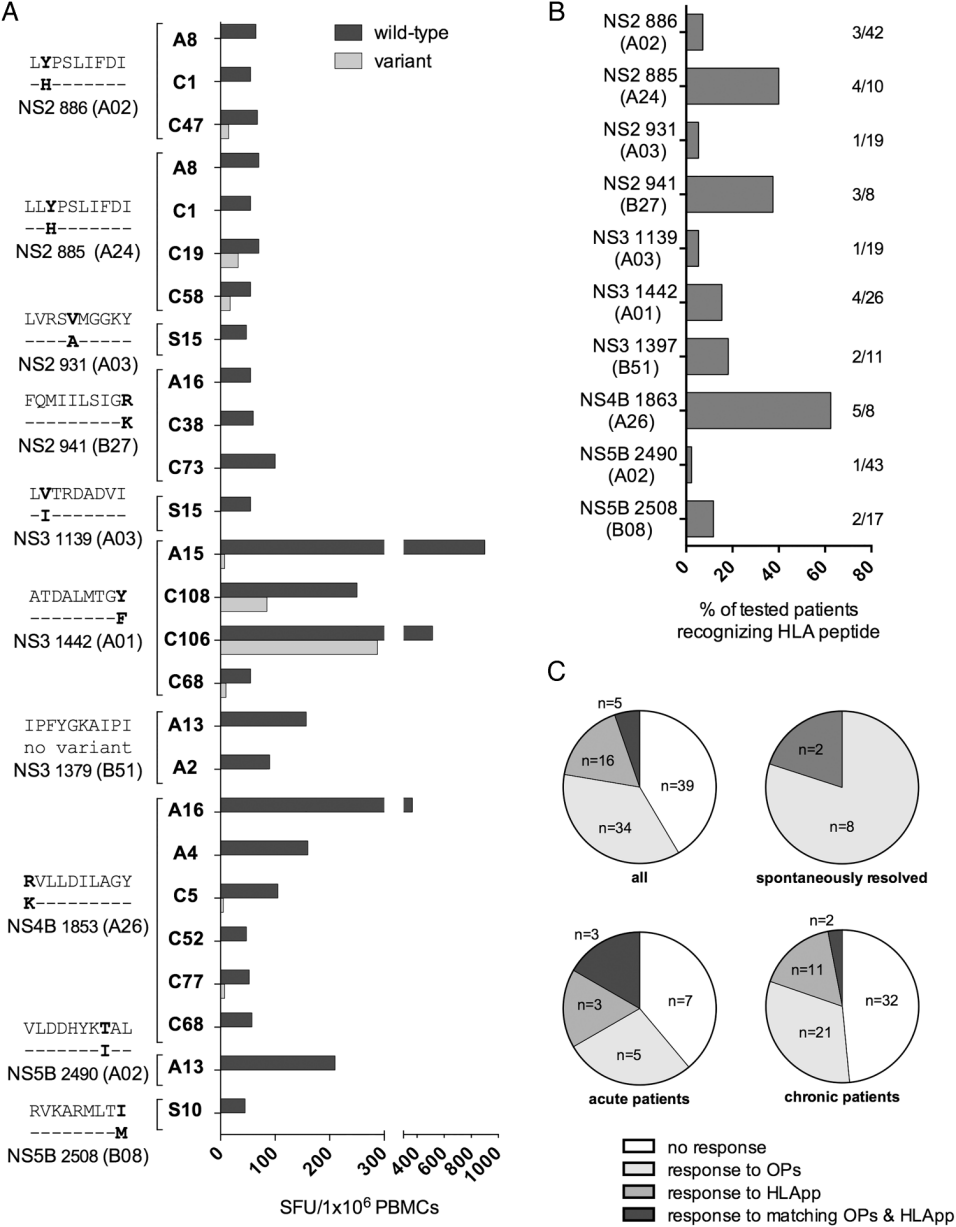
T cell responses detected using HLA-predicted peptides in HCV genotype-3 infected patients. (A) HCV genotype-3 specific T cell responses measured by IFNγ-ELISpot assays (spot-forming units (SFUs)/10^6^ peripheral blood mononuclear cells (PBMCs)) using an HLA predicted peptide set. The magnitude of T cell responses to HLA predicted wild type and variant peptides are shown. For each T cell response the responding patient, epitope wild type and variant sequence, HLA restriction and the according HCV viral region are depicted. (B) The percentage of tested HLA-matched patients mounting a detectable T cell response to HLA predicted peptides in IFNγ-ELISpot assays is depicted; also shown as number of patients responding/total number of tested HLA-positive patients. (C) Comparison of T cell responses to HLA-predicted peptides and overlapping pools (only responses to non-structural proteins) for acute, chronic and spontaneously resolved patients, performed at the level of targeted peptide pools. Responses were classified as either: no response to both peptide sets, response to either overlapping pools (OPs) or HLA predicted peptides (HLApp), response to matching OPs and HLApp. * not done; A, acute; C, chronic; SR, spontaneous resolved; HLApp, HLA predicted peptides; OPs, overlapping peptide pools; WT, wild type; V, variant; ID, patient identity number.

Recognising the fact that T cell responses may be missed using overlapping peptides,^30^ wild type and variant peptides corresponding to putative HLA class-I restricted epitopes were assessed in 88 genotype-3 patients with matching HLA types in IFNγ-ELISpot assays. Using this approach, additional T cell targets were identified in 20 patients. Overall, nine T cell epitopes were identified in four different viral regions (NS2, NS3, NS4B, NS5B) in 6/16 (37.5%) patients with acute, 12/64 (18.75%) with chronic and 2/8 (25%) patients with spontaneously resolved infection (figure 2A). Epitopes ATDALMTGY (NS3_1442_, A*01 restricted) and IPFYGKAIPI (NS3_1379_, B*51 restricted) have been previously identified in HCV genotype-1 (at positions NS3_1436_^15^ ^17^ ^20^ ^35^ ^37^ ^39^ ^40^ and NS3_1373_^17^ ^41^ ^42^). The seven remaining epitopes were novel HCV genotype-3-specific epitopes: (1) [L]LYPSLIFDI (NS2_886_; restricted by A*02 and A*24); (2) LVRSVMGGKY (NS2_931_; A*03 restricted) (3) FQMIILSIGR (NS2_941_; B*27 restricted); (4) LVTRDADVI (NS3_1139_; A*03 restricted) (5) RVLLDILAGY (NS4b_1853_; A*26 restricted); (6) VLDDHYKTAL (NS5b_2490_; A*02 restricted); and (7) RVKARMLTI (NS5b_2508_; B*08 restricted). Although T cell responses to wild type peptides were readily detected, T cell responses to the variant peptide were only detected in a single epitope (NS3_1442_) in two patients with chronic infection; these were at a lower magnitude than that made to wild type peptide (figure 2A). Viral sequence analysis in these two patients showed that the circulating HCV viral sequence was identical to the variant peptide sequence (see online supplementary table S6). T cell responses using HLA-predicted peptides were identified in the minority of patients with a matched HLA type, ranging from 2.3–40% (figure 2B), with the exception of NS4b_1853_ that was identified in 5/8 (62.5%) HLA A*26 positive patients.

Overall, using two distinct peptide-screening approaches we identified 41 distinct genotype-3 T cell targets. However, assessed at the level of peptide pools, only the minority of responses (5.7%) were detected by both approaches (see figure 2C and online supplementary figure S2) and mapped to peptide epitopes at three T cell targets (NS3_1379_, NS3_1442_ and NS5b_2490_, online supplementary table S5). Detection by both methods was highest in acutely infected patients (18.6%), whereas no overlap was observed in spontaneously resolved patients.

### T cell subset analysis and viral diversity at HCV genotype-3 T cell targets

The requirement for T cell cross-reactivity at a known target to protect against heterologous infection is dependent on the degree of viral variability at that target in the circulating viral populations. In HCV genotype-1 infection, sequence polymorphisms are more commonly observed at CD8+ compared with CD4+ epitopes.^43^ For HCV genotype-3 T cell targets defined in this study, CD4+/CD8+ subset analysis was performed at 25 targets, with 18 CD8+ and 7 CD4+ targets clearly defined. Viral sequence diversity at these targets was assessed by determining Shannon entropy scores,^44^ using an alignment of HCV genotype-3 sequences obtained from the Los Alamos sequence repository and additional inhouse sequences. Although sequence variability was higher at CD8+ than CD4+ targets (mean Shannon entropy score 0.056 vs 0.031), this did not reach statistical significance (p=0.34, see online supplementary figure S4A–H). Analysis of sequence diversity at targeted epitopes within the Oxford cohort showed more polymorphic sites relative to consensus in CD8+ compared with CD4+ epitopes (p=0.0152, see online supplementary figure S4I).

### Limited T cell cross-reactivity at HCV genotype-3 T cell targets detected in spontaneously resolved infection to viral variants found between genotypes

**Figure 3:**
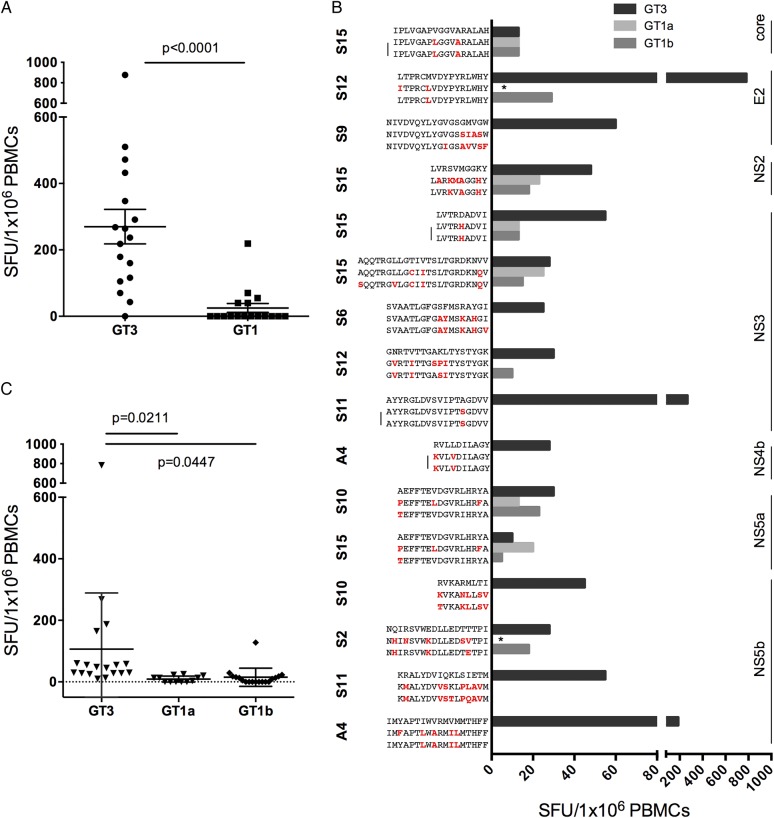
Limited cross-reactivity between HCV genotype-3 and genotype-1 in spontaneously resolved infection. HCV genotype-3 and genotype-1 specific T cell responses were measured by IFNγ-ELISpot assays (spot-forming units (SFUs)/10^6^ peripheral blood mononuclear cells (PBMC)) using (A) an HCV genotype-3 and genotype-1 specific overlapping peptide set spanning the entire HCV genome; or (B) HCV genotype-3 and genotype-1 individual peptide variants at T cell targets detected in spontaneously resolved infection. Sequence variants identical between HCV genotype-1a and 1b are marked by a bar, those not assessed by IFNγ-ELISpot assays are marked with a star. (C) Significantly reduced T cell cross-reactivity at T cell targets identified in spontaneously resolved infection was detected against common HCV genotype-1a and genotype-1b sequence variants at individual peptide level.

First, we assessed T cell cross-reactivity in patients with resolved infection using genotype-specific overlapping peptides across the whole genome. We observed that T cell responses that were almost universally present to the genotype-3 peptide pools (figure 1A) were largely absent using HCV genotype-1 peptides (see online supplementary figure S4 and figure 3A, p<0.0001).

We then determined whether HCV genotype-3-specific T cells were able to recognise common genotype-1 sequence variants at the peptide level (table 1) in spontaneously resolved patients. We used an alignment of HCV sequences obtained from the Los Alamos sequence repository and additional inhouse sequences to identify common genotype-1 sequence variants (defined as >15% of sequences) at HCV genotype-3-specific T cell targets (see online supplementary figure S5). Sequence identity between genotype-3 and genotype-1 was observed at only 1/19 T cell targets detected in spontaneously resolved infection (NS3_1379_, see online supplementary figure S5). At other T cell targets with distinct sequences between genotypes, limited cross-reactivity between genotype-1 and genotype-3 variants was observed at 15 T cell targets, tested in 16 patients with PBMC available; with reduced responses at 8 targets and no cross-reactivity at 7 targets (figure 3B, C).

**Table 1:** T cell responses detected in spontaneously resolved patients

HCV genotype-3-specific T cell response detected in spontaneous resolved patients						Number of patients responding to epitope			
Viral region	AA position	Sequence	HLA	CD4/CD8	Pept. set	S	C	A	Total
Core	27–51	GGQIVGGVYVLPRRGPRL VYVLPRRGPRLGVRATRK	ND	ND	OP	1	2	–	**3**
	143–158	IPLVGAPVGGVARALAH	ND	CD4	OPs	1	–	–	**1**
E2	610–625	LTPRCMVDYPYRLWHY	ND	ND	OPs	2	1	–	**3**
	702–719	NIVDVQYLYGVGSGMVGW	ND	CD8	OPs	2	–	1TxN	**3**
NS2	931–940	LVRSVMGGKY	A03	CD8	HLA	1	–	–	**1**
NS3	1040–1062	AQQTRGLLGTIVTSLTGR LGTIVTSLTGRDKNVV	ND	ND	OPs	1	–	–	**1**
	1139–1147	LVTRDADVI	A03	CD8	HLA	1	–	–	**1**
	1198–1213	KALQFIPVETLSTQAR	ND	ND	OPs	1	–	–	**1**
	1246–1261	KVPAAYVAQGYNVLVL	ND	ND	OPs	1	–	–	**1**
	1264–1281	SVAATLGFGSFMSRAYGI	ND	ND	OPs	1	–	1UK	**2**
	1282–1305	DPNIRTGNRTVTTGAKL GNRTVTTGAKLTYSTYGK	ND	ND	OPs	2	–	–	**2**
	1379–1387	IPFYGKAIPI	B51	CD8	HLA	–	–	1SR 1TxS	**2**
	1423–1440	AYYRGLDVSVIPTAGDVV	ND	CD4	OPs	1	1	–	**2**
	1520–1537	RPSGMFDSVVLCECYDA DSVVLCECYDAGCSWYDL	ND	CD8	OPs	2	12	–	**14**
NS4b	1805–1822	TSPLTTNQTMFFNILGGW	ND	ND	OPs	2	–	–	**2**
	1853–1862	RVLLDILAGY	A26	CD8	HLA	–	3	1TxS 1SR	**5**
NS5a	2126–2141	AEFFTEVDGVRLHRYA	ND	CD8	OPs	2	–	2TxS	**4**
NS5b	2508–2516	RVKARMLTI	B08	CD8	HLA	1	–	1TxS	**2**
	2548–2565	NQIRSVWEDLLEDTTTPI	ND	CD4	OPs	1	–	–	**1**
	2603–2618	KRALYDVIQKLSIETM	ND	CD4	OPs	1	–	–	**1**
	2844–2861	IMYAPTIWVRMVMMTHFF	ND	ND	OPs	–	–	1SR	**1**
	2893–2908	IIERLHGLSAFTLHSY	ND	CD4	OPs	1	–	–	**1**

T cell targets detected in spontaneously resolved patients and patients acutely infected who subsequently resolved infection spontaneously. For each targeted epitope, the amino acid (AA) position, peptide sequence, restricting HLA type, CD4/CD8 restriction and detecting peptide sets are specified. The total number of patients responding to the peptide and their status of infection (S, spontaneously resolved; C, chronic; A, acute; TxS, treated achieving SVR (sustained virological response); TxN, not responding to treatment; SR, spontaneously resolved) is detailed. Underlining represents amino acids that are common between overlapping peptides.

ND, not determined; OPs, overlapping peptides.

### Limited cross-reactivity within and between genotypes at dominant HCV genotype-3 T cell targets

**Figure 4:**
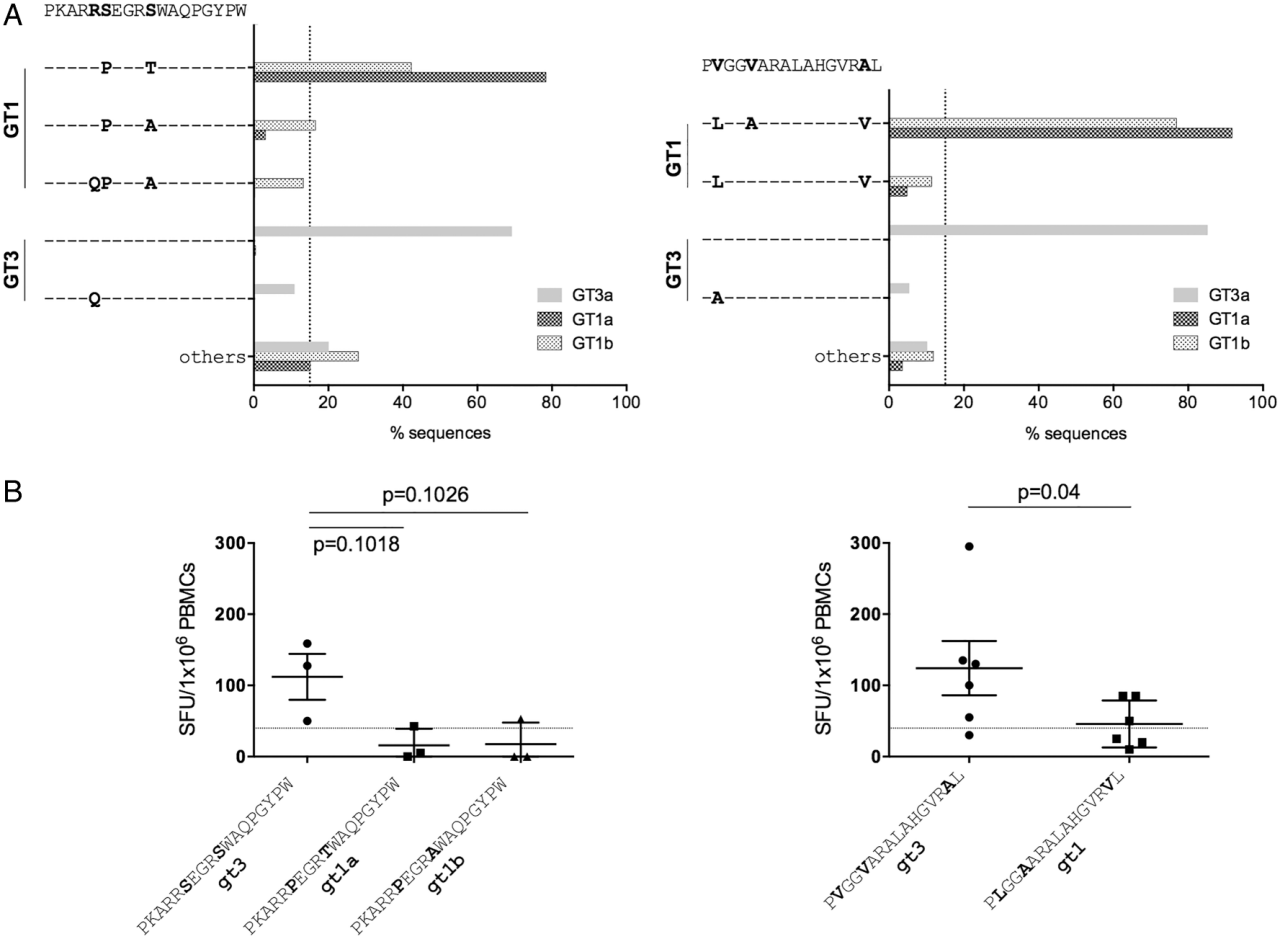
Dominant CD4+ T cell targets are variable between HCV genotypes, with limited T cell cross-reactivity against identified sequence variants. (A) HCV genotype-1 and genotype-3 sequences variants at dominant CD4+ T cell targets core_66_ and core_143_ are depicted. Sequences were obtained from the Los Alamos database, with additional HCV genotype-3 sequences generated inhouse. (B) T cell cross-reactivity as assessed by IFNγ-ELISpot assay (spot-forming units (SFUs)/10^6^ peripheral blood mononuclear cells (PBMCs)) against identified sequence variants at dominant CD4+ T cell targets core_66_ and core_143_. GT, genotype; v, variant.

**Figure 5:**
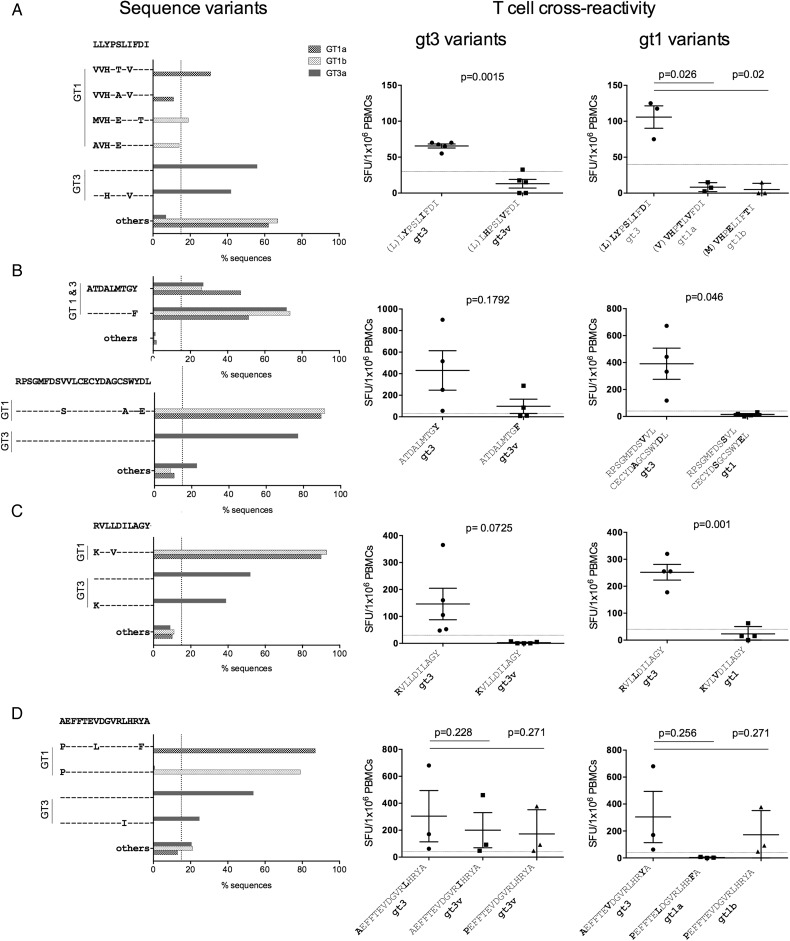
Dominant CD8+ T cell targets are variable within HCV genotype-3 and across HCV genotypes, with limited T cell cross-reactivity against identified sequence variants. (Left panel) HCV genotype-1 and genotype-3 sequence variants at dominant CD8+ T cell targets (A) NS2_886_, (B) NS3_1443_ and NS3_1520_ (C) NS4b_1853_, and (D) NS5a_2126_ are depicted. Sequences were obtained from the Los Alamos database, with additional HCV genotype-3 sequences generated inhouse. (Right panel) T cell cross-reactivity of epitope-specific T cells against identified common sequence variants at dominant CD8+ T cell targets, as assessed by IFNγ-ELISpot assays (spot-forming units (SFUs)/10^6^ peripheral blood mononuclear cells (PBMCs)) (A) NS2_886_, (B) NS3_1443_ and NS3_1520_ (C) NS4b_1853_, and (D) NS5a_2126_ is shown. GT, genotype; v, variant.

We also assessed T cell cross-reactivity against common genotype-1 and genotype-3 sequence variants at seven dominant T cell targets identified across the entire HCV genotype-3 cohort; two were CD4+ targeting HCV core, and five were CD8+ targeting HCV non-structural proteins (table 2). At the two dominant genotype-3 CD4+ T cell targets (core_66_ and core_143_) no common genotype-3 variants were identified (figure 4A). In contrast, dominant CD4+ core T cell targets varied between HCV genotypes 1 and 3 by one to three AAs (figure 3A), with limited T cell cross-reactivity detected in IFNγ-ELISpot assays (figure 4B). For the majority of CD8+ epitopes (4/5), common HCV genotype-3 sequence variants were identified, with only epitope NS3_1520_ showing a high level of conservation within genotype-3 (figure 5B, left panel). In addition, dominant CD8+ epitopes were highly divergent between HCV genotype-1 and genotype-3, with the exception of epitope NS3_1442_, which has been previously reported to be highly conserved between genotypes (figure 5B, left).^20^ Limited T cell cross-reactivity against identified HCV sequence variants was observed at all dominant CD8+ T cell targets, with reduced or abrogated recognition of common genotype-3 and genotype-1 sequence variants (figure 5, right panel).

**Table 2:** Dominant HCV genotype-3-specific T cell responses

Dominant HCV genotype-3-specific T cell response						Number of Patients responding to epitope			
Viral region	AA position	Sequence	HLA	CD4/CD8	Pept. set	S	C	A	Total
Core	66–83	PKARRSEGRSWAQPGYPW	ND	CD4	OP	–	5	–	**5**
	143–158	PVGGVARALAHGVRAL	ND	CD4	OPs	–	11	1TxN	**12**
NS2	886–896	LLYPSLIFDI	A02	CD8	HLA	–	2	1AC	**3**
		LYPSLIFDI	A24	CD8		–	3	1AC	**4**
NS3	1443–1451	ATDALMTGY	A01	CD8	HLA	–	3	1TxS	**4**
	1520–1537	RPSGMFDSVVLCECYDA DSVVLCECYDAGCSWYDL	ND	CD8	OPs	2	12	–	**14**
NS4b	1853–1862	RVLLDILAGY	A26	CD8	HLA	–	3	1TxS 1SR	**5**
NS5a	2126–2141	AEFFTEVDGVRLHRYA	ND	CD8	OPs	2	–	2TxS	**4**

Dominant T cell responses, defined as targeted in more than four patients within the Oxford HCV genotype-3 cohort, are depicted. For each targeted epitope, the amino acid (AA) position, peptide sequence, restricting HLA type, CD4/CD8 restriction and detecting peptide sets are specified. The total number of patients responding to the peptide and their status of infection (S, spontaneously resolved; C, chronic; A, acute; AC, acute proceeding to chronic; TxS, treated achieving SVR (sustained virological response); TxN, not responding to treatment; SR, spontaneously resolved) is detailed.

Underlining represents amino acids that are common between overlapping peptides. ND, not determined; OPs, overlapping peptides.

### T cell specificity is distinct between HCV genotypes 1 and 3 infection across the HCV genome

**Figure 6:**
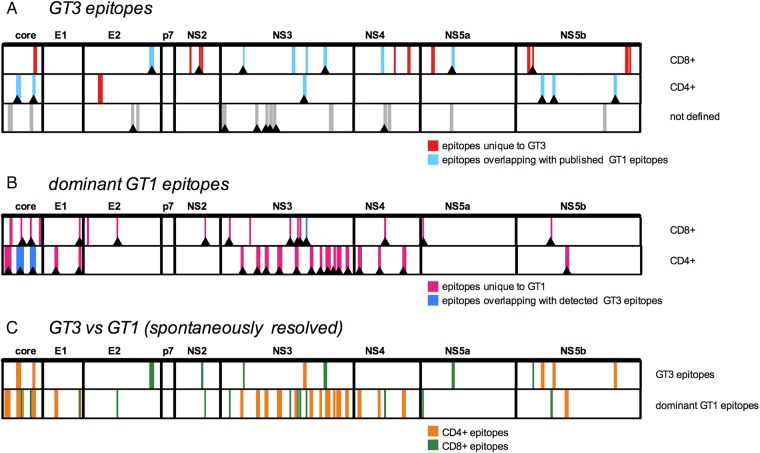
T cell targets are distinct in HCV genotypes 1 and 3. Comparison of T cell specificity in HCV genotypes 1 and 3: (A) HCV genotype-3 CD4+ and CD8+ T cell targets and those without defined CD4/CD8 restriction described in this study are depicted. Genotype-3 T cell targets previously described/not described in HCV genotype-1 infection (as deposited on the immune epitope database, IEDB) are colour coded in light blue/red, respectively. T cell targets detected in at least one patient with spontaneously resolved infection are marked with an arrow. (B) Dominant HCV genotype-1 epitopes as derived from the IEDB are depicted; those detected/not detected in HCV genotype-3 in this study are colour coded in blue/pink, respectively. Genotype-1 T cell targets identified in patients with spontaneously resolved infection in the literature are marked with an arrow. (C) Comparison of HCV immunogenic regions in HCV genotype-3 infection (identified in this study) and HCV genotype-1 infection (from IEDB) that were targeted in patients with spontaneously resolved infection. CD4+ (orange) and CD8+ (green) T cell targets are colour coded. GT, genotype.

Finally, the overlap in T cell specificity between HCV genotypes 1 and 3 was evaluated across the viral genome. For HCV genotype-1, previously described T cell targets were obtained from the immune epitope database (http://www.iedb.org/) and these were aligned with HCV genotype-3 T cell targets detected in the Oxford cohort for comparison (see figure 6A and online supplementary tables S7 and S8). The majority (11/18) of HCV genotype-3-specific CD8+ T cell targets did not overlap with epitopes previously described in genotype-1. However, six out of seven HCV genotype-3-specific CD4+ epitopes overlapped with those previously described in HCV genotype-1 infection. Next, dominant published HCV genotype-1 epitopes were compared with the Oxford HCV genotype-3 T cell targets (see figure 6B and supplementary figure S6). Minimal overlap in T cell specificity was found at 18 CD8+ epitopes dominant in HCV genotype-1 infection: only one epitope overlapped with those detected in the Oxford HCV genotype-3 cohort. Similarly, of 20 HCV regions frequently targeted by CD4+ cells in HCV genotype-1 infection, overlapping T cell responses in HCV genotype-3 infection were only detected in 2 cases. Overall, T cell specificity was markedly different between HCV genotypes in patients with resolved infection (figure 6C).

## Discussion

To date, the assessment of T cell immunity in HCV has focused on HCV genotype-1 infection since this infection is dominant in wealthy countries, and was historically more difficult to treat. However, globally more than 53 million people are infected with genotype-3, and in the era of DAA therapy genotype-3 is more difficult to treat.^45^ ^46^ This means that the evaluation of T cell immunity in genotype-3 with a view to developing vaccines capable of targeting multiple genotypes is increasingly relevant. In this study, we set out to perform a comprehensive assessment of T cell specificity in a large cohort of HCV genotype-3 infected patients with acute, resolved and chronic HCV infection. In addition we assessed T cell cross-reactivity with common genotype-3 and genotype-1 viral variants focusing particularly on people with resolved infection, where T cell induction has been shown to play a critical role in viral control. Overall, we show that only the minority of T cell targets is recognised by both genotypes, and that cross-reactivity between common circulating genotype-1 and genotype-3 viral sequence variants is limited.

Similar to published data for HCV genotype-1, we show that T cell responses are readily detectable in resolved infection using genotype-3 specific peptides, target multiple HCV antigenic regions and are of a higher magnitude compared with people with chronic disease, where responses are undetectable in approximately 50% of people^35^ ^47–49^ We also observed that overall, patients with resolved genotype-3 infection preferentially targeted HCV non-structural proteins. Together, this data suggests that T cells, particularly to the non-structural regions play an important role in viral clearance irrespective of the viral genotype and that early maintenance of this response is important in viral control.

The detailed assessment of T cell specificity revealed notable differences with limited cross-reactivity between HCV genotypes 1 and 3; to assess T cell specificity we used overlapping peptides in pools derived from a genotype-3 sequence spanning the entire HCV genome. In addition we used a sequence-based screening approach to identify putative HLA-class-I epitopes through the prior assessment of polymorphic HCV genomic sites associated with host HLA in a large cohort of patients with HCV genotype-3 infection.^16^ The advantage of the latter approach is that the optimal epitope length, HLA restriction, and functionally relevant ‘escape’ peptide variants linked to HLA associated T cell escape are predefined. However, this approach is dependent on bioinformatic analysis with a reduced capacity to identify epitopes restricted by rare HLA alleles where information of HLA/peptide binding may be lacking, and by necessity will only identify epitopes where viral variation as a result of T cell pressure occurs. In contrast, overlapping peptides allow for the detection of T cell epitopes across the entire genome in regions where viral escape does not or cannot occur, but that nevertheless may play an important role in viral control. These two approaches were complementary and together identified 41 distinct CD4+ and CD8+ T cell targets in HCV genotype-3.

The requirement for T cell cross-reactivity at a known target to protect against heterologous infection either in natural infection, or following vaccination is dependent on the degree of viral variability at that target in the circulating viral population. At a population level, the majority of T cell targets were not conserved within genotype-3, or between genotype-1 and genotype-3. An analysis of the viral diversity at targeted epitopes within our cohort showed more variability within CD8+ compared with CD4+ targets, consistent with published longitudinal data showing that viral escape to CD4+ epitopes is relatively unusual.^43^

We assessed T cell cross-reactivity in patients with resolved infection first using genotype-specific overlapping peptides and showed that cross-reactivity was minimal. However, we also found that T cell cross-reactivity was absent or reduced when assessed at a peptide level using common circulating genotype-1 peptide variants. Similarly, there was minimal evidence of T cell cross-reactivity when we assessed dominant genotype-3 responses among the whole cohort. This is in line with previously published cross-reactivity data at dominant HCV genotype-1 epitopes.^21^ We cannot exclude the possibility that some of the patients in our cohort were infected with multiple HCV genotypes. New next generation sequencing technologies currently in development may improve the resolution in detecting mixed genotype infection. Nevertheless this is not expected to impact on our measurement of T cell cross-reactivity ex vivo.

Finally, we show that T cell specificity across the HCV genome differs between HCV genotypes 1 and 3, including people with resolved genotype-1 and genotype-3 infection consistent with previous results reporting substantial differences in the patterns of viral adaptation to HLA-restricted immune pressure^16^ and differences in T cell responses to the NS3 region between HCV genotypes 1 and 3.^17^ In contrast, a recent study analysing responses in HCV genotype-1 and genotype-4 infection suggested that similar HCV regions are targeted in these genotypes, however, responses were not mapped to epitope level.^50^

HCV sequence diversity is thought to be one of the major obstacles in the development of an effective vaccine. Currently HCV T cell vaccines have completed phase-I assessment and are now in phase-IIb efficacy testing.^15^ ^51^ In these studies we have shown that HCV vaccines based on simian adenoviral vectors encoding an HCV genotype-1b strain shown some cross-reactivity (approximately 30%) to non-genotype-1 HCV. Parallel efforts in the development of B cell vaccines that aim to induce cross-protective neutralising antibodies against the HCV envelope in distinct viral genotypes are also underway.^52^ To date, HCV sequence diversity has been rarely taken into account in the design of HCV immunogens for prophylactic vaccines; a single study specifically aiming to induce cross-reactive T cell responses has assessed the ability of HCV genotype-1 ancestral and consensus sequences to prime T cell immune responses,^53^ and we have recently published an in vivo priming model that seeks to identify T cell variants that are maximally cross-reactive for inclusion into a HCV immunogen.^54^

Future HCV immunogens that aim to target multiple genotypes may need to focus on new approaches to target multiple HCV genotypes to generate vaccines that are applicable in settings where mixed genotypes circulate in the population. This may be possible using viral vectored strategies that can encode large immunogens.^15^ Some approaches that are currently in development for vaccines against immunodeficiency virus may be readily also applied to HCV. This may include vaccines encoding viral regions that are conserved between genotypes,^29^ excluding variable epitopes dominant in natural infection, with the hope of inducing T cells to subdominant epitopes. Alternative approaches include the use of multivalent mosaic immunogens that encode antigens derived from multiple genotypes.^55^

In conclusion, we show that HCV T cell specificity is distinct between two highly prevalent global genotypes with limited T cell cross-reactivity between common viral variants at dominant epitopes. Since this also holds true for people with resolved infection, our data suggests that regions frequently targeted in natural HCV infection may not serve as attractive targets for a vaccine that aims to protect against multiple HCV genotypes.

## Supplementary Material

Web supplement
