# Supplementary material for: The broad assessment of HCV genotypes 1 and 3 antigenic targets reveals limited cross-reactivity with implications for vaccine design
Source: Gut. 2015 Jun 19;65(1):112–23. doi: 10.1136/gutjnl-2014-308724 (PMC4717358; doi:10.1136/gutjnl-2014-308724)
Supplement: Web supplement [file gutjnl-2014-308724-s1.pdf]

		spontaneous resolvers		acutely infected		chronic patients	
number of patients		16	%	16	%	108	%
sex	female	5	31.3	7	43.8	31	28.7
	male	11	68.8	9	56.3	77	71.3
Age	>40	7	43.8	5	31.3	35	32.4
	<40	9	56.3	11	68.8	73	67.6
Ethnicity	Caucasian	16	100.0	15	93.8	87	80.6
	Asian					16	14.8
	Hispanic			1	6.3	1	0.9
	not known					4	3.7
Risk factor	IVDU	11	68.8	9	56.3	70	64.8
	unkown	1	6.3			14	13.0
	BP			1	6.3	12	11.1
	cocaine					6	5.6
	tattoo	2	12.5	1	6.3	3	2.8
	needlestick	1	6.3			2	1.9
	sexual	1	6.3	5	31.3	0	0.0
	vertical					1	0.9
HCV subtype	Genotype 3		N/A	8	50.0	14	13.0
	Genotype 3a		N/A	8	50.0	94	87.0
Viral load	IU/ml (mean)		N/A		1827490		872828
	IU/ml (range)		N/A		6290-8930000		3614-17880410
Outcome	cleared spontaneously	16	100.0	4	25.0	0	0.0
	chronic	0	0.0	10	62.5	108	100.0
	not treated	N/A		6	60.0	30	27.8
	treated	N/A		4	40.0	78	72.2
	unkown			2	12.5		
Treatment	SVR			2	50.0	42	53.8
	REL			1	25.0	25	32.1
	NR			1	25.0	6	7.7
	incomplete					3	3.8
	not known					2	2.6

Table S1: Patient details of patients with spontaneously resolved, acute and chronic HCV genotype-3 infection.
ID: patient identifier; IVDU: intravenous drug use; BP: blood products; SVR: sustained virological response; REL: viral relapse; NR: treatment non-response.

ID	Age	Sex	Race	Risk factor	GT	HCV VL	ALT	Treatment	outcome
						Iu/ml			
A1	23	F	W	Sexual	3	>700000	68	naïve	cleared spontaneously
A2	31	M	W	IVDU	3a	6290	not known	naïve	cleared spontaneously
A3	23	M	W	IVDU	3a	8930000	46	naïve	cleared spontaneously
A4	21	M	W	IVDU	3	15900	72	naïve	cleared spontaneously
A5	19	M	W	IVDU	3	168000	229	naïve	chronic
A6	20	F	W	IVDU	3	16100	38	naïve	chronic
A7	46	M	W	blood exposure	3	204000	592	naïve	chronic
A8	28	M	W	Sexual	3	136000	698	naïve	chronic
A9	45	F	W	Tattoo	3a	>700000	201	naïve	chronic
A10	21	F	W	IVDU	3a	600000	not known	naïve	chronic
A11	18	F	W	IVDU	3	102000	88	naïve	lost in <6 months
A12	25	F	W	IVDU	3a	not known	21	naïve	lost in <6 months
A13	40	F	W	Sexual	3a	>700000	464	treated	cleared on Tx
A14	41	M	H	Sexual	3a	67535	250	treated	cleared on Tx
A15	49	M	W	Sexual	3a	46603	1441	treated	cleared on Tx
A16	20	M	W	IVDU/Tattoo	3	>700000	383	treated	chronic (non-responder)

Table S2: Patient details acute HCV genotype-3 cohort.
ID: patient identifier; M: male; F: female; W: white; H: Hispanic; IVDU: intravenous drug use; GT: genotype; ALT: alanine aminotransferase; Tx: treatment.

Protein	HLA	Wildtype	Variant	Amino Acids	Peptide ID	Predicted Epitope
NS2	A0101	V	A	879-887	001-A0101	**V**ILLTSLLY
	A02	L	P	862-871	049-A02	ALQVWVPPL**L**
		L	P	870-879	048-A02	L**L**ARGSRDGV
		Y	H	881-890	052-A02	LLTSLL**Y**PSL
		Y	H	885-894	047-A02	LL**Y**PSLIFDI
	A0301	V	A	926-935	006-A0301	RLCMLVRS**V**M
		V	A	929-938	004-A0301	MLVRS**V**MGGK
		V	A	930-939	005-A0301	LVRS**V**MGGKY
	A2402	Y	H	886-894	007-A2402	L**Y**PSLIFDI
	B1501	T	A	878-887	008-B1501	GVILL**T**SLLY
	B15	I	V	946-954	055-B15	S**I**GRWFNTY
	B2705	R	K	940-949	010-B2705	FQMIILSIG**R**
		R	K	948-956	009-B2705	G**R**WFNTYLY
		H	Y	962-971	011-B2705	MQ**H**WAAAGLK
	B4402	I	V	822-831	012-B4402	ATLGAG**I**LVL
	B44	I	V	826-835	057-B44	AG**I**LVLFGFF
	B5101	S	G	871-880	013-B5101	LARG**S**RDGVI
	C03	L	M	829-838	050-C03	I**L**VLFGFFTL
	C04	V	I	981-990	051-C04	IFSPMEIK**V**I
NS3	A0101	Y	F	1442-1450	014-A0101	ATDALMTG**Y**
	A0201	A	D	1389-1398	015-A0201	**A**LLKGGRHLI
	A0301	V	I	1138-1146	016-A0301	L**V**TRDADVI
	B1501	K	R	1296-1305	017-B1501	**K**LTYSTYGKF
	B2705	L	I	1379-1388	018-B2705	IPFYGKAIP**L**
		V	I	1632-1641	019-B2705	YRLGP**V**QNEI
	B4402	G	S	1407-1416	020-B4402	DEIASKLR**G**M
	B4403	L	S	1639-1647	021-B4403	NEIC**L**THPI
	B5101	A	D	1388-1397	023-B5101	I**A**LLKGGRHL
NS4B	A02	A	T	1873-1882	062-A02	KIMGGELPT**A**
		A	T	1880-1889	065-A02	PT**A**EDMVNLL
	A0301	I	V	1901-1910	026-A0301	GV**I**CAAILRR
	A2601	R	K	1852-1861	027-A2601	**R**VLLDILAGY
	A68	A	I	1736-1744	067-A68	EK**A**LGLLQR
	B27	R	K	1948-1957	061-B27	A**R**VTALLSSL
	B4001	I	V	1847-1855	028-B4001	G**I**GLGRVLL
	B51	A	T	1879-1888	066-B51	LPT**A**EDMVNL
		A	T	1881-1889	064-B51	T**A**EDMVNLL
	C0401	V	I	1733-1742	025-C0401	QFKEK**V**LGLL
NS5A	A0201	G	S	2321-2330	029-A0201	ALPPR**G**APPV
	A0301,	V, P	A, S	2382-2391	030-A0301	K**V**PPS**P**GGES
	A2601	D	G	2268-2276	033-A2601	ET**D**AELSVA
	A68	T	S	1989-1998	070-A68	WVC**T**VLSDFK
	B0702	V	E	2309-2317	036-B0702	APDY**V**PPTV
		V	E	2313-2322	035-B0702	**V**PPTVHGCAL
		T	A	2332-2341	034-B0702	PPRRKR**T**IQL
	B0801	I	V	2251-2259	037-B0801	ESETKVV**I**L
	B44	R	Q	2097-2105	071-B44	VEVR**R**VGDF
NS5B	A0201	T	I	2489-2498	038-A0201	VLDDHYK**T**AL
		N	D	2540-2549	040-A0201	SLSSKAI**N**QI
		N	D	2544-2552	039-A0201	KAI**N**QIRSV
	A1101	R	K	2500-2509	041-A1101	EVKERAS**R**VK
	A2601	K	R	2537-2545	042-A2601	DVRSLSS**K**A
	B0801	I	M	2507-2515	043-A2601	RVKARMLT**I**
	B1501	Q	L	2476-2484	044-B1501	S**Q**RQKKVTF
	B5101	K	R	2474-2482	045-B5101	SASQRQ**K**KV

Table S3: HLA-associated polymorphisms – sequence polymorphisms located *within* the predicted epitope sites.
HCV viral region, associated HLA type, epitope position, peptide identifier and sequence, as well as wild-type and variant sequences are depicted. Polymorphic sites associated with patient HLA are marked in in bold.

Protein	HLA	Wildtype	Variant Residue	Amino Acids	Polymor-phic Site	Peptide ID	Predicted Epitope and Wild-type Residue ()
NS2	A0201	I	V	934-942	943	002-A0201	VMGGKYFQM(**I**)
		I	V	944-953	943	003-A0201	(**I**)ILSIGRWFNT
	A02	L	P	861-870	871	054-A02	AALQVWVPPL(**L**)
		Y	H	878-886	887	053-A02	GVILLTSLL(**Y**)
NS3	A68	L	F	1047-1056	1046	058-A68	(**L**)GTIVTSLTGR
	B5101	A	D	1379-1388	1389	022-B5101	IPFYGKAIPI(**A**)
		A	D	1390-1398	1389	024-B5101	(**A**)QLKGGRHLI
NS5A	A1101	V	A	2374-2382	2383	031-A1101	DTQSSTTSK(**V**)
	A2402	D	E	2138-2147	2148	032-A2402	RYAPPCKPLL(**D**)
NS5B	B5101	M	L	2846-2854	2855	046-B5101	APTIWVRMV(**M**)

Table S4: HLA-associated polymorphisms – sequence polymorphisms located *flanking* the predicted epitope sites.
HCV viral region, associated HLA type, epitope position, peptide identifier and sequence as well as wild-type and variant sequences are depicted. Polymorphic sites flanking epitope are indicated in bold within brackets ().

Protein	Position	3a peptide sequence	Patient		Patient viral sequence	CD8/CD4
core	27-51	GGQIVGGVYVLPRRGPRL	C58	*	GGQIVGGVYVLPRRGPRL	N/A
		VYVLPRRGPRLGVRATRK	C106		VYVLPRRGPRLGVRATRK	
			S11		SR	
	66-90	PKARRSEGRSWAQPGYPW	C5		ND	CD4
		GRSWAQPGYPWPLYGNEG	C12		PKARRSEGRSWAQPGYPW	
			C37		PKARRSEGRSWAQPGYPW	
			450		PKARRSEGRSWAQPGYPW	
			C68	*	GRSWAQPGYPWPLYGNEG	
	130-147	FADLMGYIPLVGAPVGGV	C15		FADLMGYIPLVGAPVGGV	N/A
			C5		ND	
	137-154	IPLVGAPVGGVARALAH	S15		SR	N/A
	143-158	PVGGVARALAHGVRAL	A16		PVGGVARALAHGVRAL	CD4
			C6	*	P**A**GGVARALAHGVRAL	
			C12		PVGGVARALAHGVRAL	
			C13		PVGGVARALAHGVRAL	
			C18		PVGGVARALAHGVRAL	
			C19		PVGGVARALAHGVRAL	
			C22		PVGGVARALAHGVRAL	
			C23		PVGGVARALAHGVRAL	
			C27		PVGGVARALAHGVRAL	
			331	*	PVGGVARALAHGVRAL	
			C70		PVGGVARALAHGVRAL	
			C77	*	PVGGVARALAHGVRAL	
	148-165	ARALAHGVRALEDGINFA	C103		ARALAHGVRALEDGINFA	CD8
E2	460-476	CKPITFFRQGWGSLTDA	A15		CKPITFFRQGWGSLTDA	CD4
		FRQGWGSLTDANITGPSD	C106		F**N**QGWGSLTDANI**S**GPSD	
	610-625	LTPRCMVDYPYRLWHY	C106		LTPRC**L**VDYPYRLWHY	N/A
			S1	*	SR	
			S12		SR	
	635-650	KVRMFVGGFEHRFTAA	A11		KVRMFVGGFEHRFTAA	N/A
			C13		KVRMFVAGFEHRFTAA	
	696-719	LIHLHQNIVDVQYLYGV	S4	*	SR	CD8
		NIVDVQYLYGVGSGMVGW	A16		NIVDVQYLYGVGSGMVGW	
			S9	*	SR	

Table S5: HCV genotype-3-specific T-cell targets in HCV structural regions.
HCV genotype-3-specific T-cell targets identified using overlapping peptide pools in HCV structural regions are depicted. All patients targeting specific individual peptides are specified (colour coding: orange-SR, blue-acute, yellow chronic). Circulating viral sequence is depicted when obtained, as well as CD4+ /CD8+ subset analysis. Sequence polymorphisms differing from overlapping peptide set sequence are marked in red. * Epitopes previously described in (Humphreys et al. 2012). N/A not available.

Protein	Position	3a peptide sequence		Patient		Patient viral sequence	CD8/CD4
NS2	886-896	(L)LYPSLIFDI	#	A8		LLYPSLIFDI	CD8
			#	A8		LYPSLIFDI	
			#	C1		LYPSLIFDI	
			#	C19		LYPSLIFDI	
			#	C47		LYPSLIFDI	
			#	C58		LYSSLIFDI	
	931-940	LVRSVMGGKY	#	S15		SR	CD8
	941-951	FQMIILSIGR	#	A16		FQMAILSIGR	CD8
			#	C38		FQMIILGVGR	
			#	C73		FQMVILSIGK	
NS3	1040-1062	AQQTRGLLGTIVTSLTGR		S15		SR	N/A
		LGTIVTSLTGRDKNVV					
	1139-1147	LVTRDADVI	#	S15		SR	CD8
	1198-1213	KALQFIPVETLSTQAR		S5	*	SR	N/A
	1246-1261	KVPAAYVAQGYNVLVL		S8	*	SR	N/A
	1264-1281	SVAATLGFGSFMSRAYGI		A11		SVAATLGFGSFMSHAYGI	N/A
				S6		SR	
	1282-1305	DPNIRTGNRTVTTGAKL		S1	*	SR	N/A
		GNRTVTTGAKLTYSTYGK		S12		SR	
	1370-1387	EEVALGSEGEIPFYGKAI		A13		ND	CD8
	1379-1387	IPFYGKAIPI	#	A13		ND	
			#	A2		ND	
	1423-1440	AYYRGLDVSVIPTAGDVV		C22		AYYRGLDVSVIPTAGDVV	N/A
				S11	*	SR	CD4
	1436-1447	GDVVVCATDALMTGF		A15		GDVVVCATDALMTGY	CD8
	1442-1447	ATDALMTGY	#	A15		ATDALMTGY	
			#	C68		ATDALMTGF	
			#	C106		ATDALMTGF	
			#	C108		ATDALMTGF	
	1520-1537	RPSGMFDSVVLCECYDA		C5		ND	CD8
		DSVVLCECYDAGCSWYDL		C7		RPSGMFDSVVLCECYDAGCSWYDL	
				C15	*	RPSGMFDSVVLCECYDAGCSWYDL	
				C17		RPSGMFDSVVLCECYDAGCSWYDL	
				C19		RPSGMFDSVVLCECYDAGCSWYDL	
				C27	*	RPSGMFDSVVLCECYDAGCAWYDL	
				C35		ND	
				C42		ND	
				C44		RPSGMFDSVVLCECYDAGCSWYDL	
				C58	*	RPSGMFDSVVLCECYDAGCSWYDL	
				C96		ND	
				C98		ND	
				S13		SR	
				S14		SR	
	1547-1569	RAYLSTPGLPVCQDHLDF		C19		RAYLSTPGLPVCQDHLDFWESVF	N/A
		GLPVCQDHLDFWESVF					
NS4B	1792-1808	PAVASLMAFTASVTSPL		A8		ND	CD8
				C68	*	PAVASLMAFTASVTSPL	
				C77	*	ND	
	1805-1822	TSPLTTNQTMFFNILGGW		S8	*	SR	N/A
				S13		SR	
	1825-1842	THLAGPQSSSAFVVSGLA		C77	*	ND	N/A
	1853-1862	RVLLDILAGY	#	A16		KVLLDILAGY	CD8
			#	A4		ND	
			#	C5		KVLLDILAGY	
			#	C52		ND	
			#	C77		ND	
	1917-1932	EGAVQWMNRLIAFASR		C67	*	EGAVQWMNRLIAFASR	CD8
NS5A	2030-2047	GVMSTRCPCGASIAGHVK		C13	*	GVMSTRCPCGASITGHVK	CD8
	2119-2136	CPCQVPAAEFFTEVDGVR		A8		CPCQVPAPEFFTEVDGVR	
	2126-2141	AEFFTEVDGVRLHRYA		A13		ND	CD8
				A15		ND	
				S10		SR	
				S15		SR	
	2145-2162	KPLLRDEITFMVGLNSYA		A5		ND	N/A
NS5B	2484-2499	TFDRLQVLDDHYKTAL		A13		TFDRLQVLDDHYKTAL	CD8
	2490-2499	VLDDHYKTAL	#	C68		ND	
	2508-2516	RVKARMLTI	#	A13		RVKARMLTI	CD8
			#	S10		SR	
	2548-2565	NQIRSVWEDLLEDTTTPI		S2	*	SR	CD4
	2603-2618	KRALYDVIQKLSIETM		S11	*	SR	CD4
	2844-2861	IMYAPTIWVRMVMMTHFF		A4		ND	N/A
	2893-2908	IIERLHGLSAFTLHSY		S2	*	SR	CD4
	2947-2964	GKAKICGLYLFNWAVRTK		C37	*	GKAKITGLYLFNWAVRTK	CD8
	2967-2976	KLTPLPAAGQL		450	*	KLTPLPAAGLL	CD8

Table S6: HCV genotype-3-specific T-cell targets in HCV non-structural regions. HCV genotype-3-specific T-cell targets identified using overlapping peptide pools and HLA predicted peptides (#) in HCV structural regions are depicted. All patients targeting specific individual peptides are specified (colour coding: orange-SR, blue-acute, yellow chronic). Circulating viral sequence is depicted when obtained, as well as CD4+ /CD8+ subset analysis. Sequence polymorphisms differing from overlapping peptide set sequence are marked in red. *Epitopes previously described in (Humphreys et al. 2012). N/A not available.

GT3 CD8+ epitopes (Oxford Study Cohort)						GT1 CD8+ epitopes in corresponding regions		
Position	3a peptide sequence	Viral protein	HLA	Pt ID	HLA type	Peptide (Literature)	HLA	First author
931-940	LVRSVMGGKY	NS3	A03	S15	A*3201 A*0301 B*1401 B*0702 C*0802 C*0702 #	no CD8 epitopes described		
941-951	FQMIILSIGR	NS2	B27	A16	A*0201 A*2601 B*2702 B*3801 C*1203 #	no CD8 epitopes described		
				C38	A*0201 A*1101 B*1801 B*2705 C*0102 C*1203 #			
				C73	A*0201 B*5701 B*2702 C*0701 C*1501 #			
1917-1932	EGAVQWMNRLIAFASR	NS4B		C67	A*0101 A*3001 B*1302 B*4402 C*0602 C*0501 # *	no CD8 epitopes described		
2030-2047	GVMSTRCPCGASIAGHVK	NS5A		C13	A*1101 A*7401 B*4403 B*38 C*04 C*0702 # *	no CD8 epitopes described		
2484-2499	TFDRLQVLDDHYKTAL	NS5B		A13	A*0101 B*0801 B*5101 C*0102 C*0701 #	no CD8 epitopes described		
2490-2499	VLDDHYKTAL		A02	C68	A*0101 A*0201 B*0801 B*5701 C*0602 C*0701 #			
2508-2516	RVKARMLTI	NS5B	B08	A13	A*0101 B*0801 B*5101 C*0102 C*0701 #	no CD8 epitopes described		
				S10	A*3201 A*0101 B*0801 B*4402 C*0501 C*0701			
2967-2976	KLTPLPAAGQL	NS5B		450	# *	no CD8 epitopes described		
1853-1862	RVLLDILAGY	NS4B	A26	A16	A*0201 A*2601 B*2702 B*3801 C*120301 #	ILAGYGAGV	A2	M Battegay
				A4	A*0201 A*2601 B*3801 B*4402 C*0501 C*1203 #	ILAGYGAGV	A2	N H Gruener
				C6	A*2301 A*2601 B*3801 B*4901 C*0701 C*1203 #	ILAGYGAGV	A2	T Kuntzen
				C52	A*0101 A*2601 B*4501 B*0801 C*0602 C*0701 #			
				C77	A*0201 A*2601 B*3801 B*2702 C*1203 C*0102 #			
886-896	LLYPSLIFDI/LYPSLIFDI	NS2	A02/A24	A8	A*2402 A*0201 B*3502 B*4403 C*0401 C*1601 #	**H**P**T**L**V**FDITK	Class I	A L Cox
				C1	A*1101 A*2404 B*18 B*3501 C*0401 C*1203 #	**H**P**T**L**V**FDITKL	Class I	T Kuntzen
				C19	A*24 B*3501 B*4403 C*0401 C*0409 #			
				C47	A*0205 A*2402 B*1302 B*4901 C*0602 C*0701 #			
				C1	A*0205 A*2402 B*1302 B*4901 C*0602 C*0701 #			
				C58	A*0101 A*0201 B*0702 B*4001 C*0304 C*0702 #			
2947-2964	GKAKICGLYLFNWAVRTK	NS5B		C37	A*1101 B*0702 B*4402 C*0501 C*0702 # *	RGG**R**A**A**ICG**K**YLFNWAVR	Class I	C Neumann-Haefelin
						G**R**A**A**ICG**K**Y	B27	C Neumann-Haefelin
						G**R**A**A**ICG**K**YLFNWAV	Class I	P T F Kennedy
						**K**YLFNWAV**K**	A2	Z Guo
148-165	ARALAHGVRALEDGINFA	core		C103	#	GVR**V**LEDG**V**	A2	H F Löhr
						R**V**LEDGVN**Y**	Class I	D D Anthony
						**V**LEDGVN**Y**ATGNLPG	Class I	K Sugimoto
1139-1147	LVTRDADVI	NS3	A03	975	A*3201 A*0301 B*1401 B*0702 C*0802 C*0702 #	VTR**H**ADVIPV	Class I	T Kuntzen
1520-1537	RPSGMFDSVVLCECYDAGCSWYDL	NS3	B35/C04	C5	A*1101 A*1101 B*3501 B*5101 C*0401 C*1402	MFDS**S**VLCECYDAGC	Class I	D Ciuffreda
				C7	A*0101 A*0201 B*35 B*14 C*0401 C*0802			
				C15	A*2402 A*3002 B*0702 B*3501 # *			
				C17	A*1101 A*1101 B*1517 B*3501 C*0401 C*0701			
				C19	A*1101 A*2402 B*18 B*3501 C*0401 C*1203			
				C27	A*0301 A*3002 B*0702 B*3501 C*0401 C*0702 # *			
				C35				
				C42	A*0101 A*2901 B*3501 B*4403 C*0401 C*1601			
				C44	A*0301 A*2402 B*3501 B*5501 C*0301 C*0401			
				C58	A*2402 B*3501 B*4403 C*0401 C*0409 # *			
				C96				
				C98	A*0201 A*1101 B*3501 B*4001 C*0401 C*0301			
				S13	A*0301 A*2601 B*3501 B*2701 C*0401 C*0202			
				S14	A*0201 A*1101 B*4402 B*3501 C*0401 C*0501			
2126-2141	AEFFTEVDGVRLHRYA	NS5A		A13	A*0101 B*0801 B*5101 C*0102 C*0701 #	FFTE**L**DGVRLHRFAP	Class I	D Ciuffreda
				A15	A*0101 B*5201 B*5701 C*0602 C*1202			
				S10	A*3201 A*0101 B*0801 B*4402 C*0501 C*0701			
				S15	A*3201 A*0301 B*1401 B*0702 C*0802 C*0702			
1792-1808	PAVASLMAFTASVTSPL	NS4B		6-23	A*2402 A*0201 B*3502 B*4403 C*0401 C*1601	SLMAFTA**A**V	A2	B Rehermann
				C68	A*0101 A*0201 B*0801 B*5701 C*0602 C*0701 # *	SLMAFTA**A**V	A2	K M Chang
				C77	A*0201 A*2601 B*3801 B*2702 C*1203 C*0102 # *	SLMAFTA**A**V	A2	N H Gruener
696-719	LIHLHQNIVDVQYLYGVGSGMVGW	E2		S4	A*02 A*3201 B*2707 B*1501 C*0602 C*0304 # *	ALSTGLIHLHQNIVD	Class I	D Ciuffreda
				A16	A*0201 A*2601 B*2702 B*3801 C*1203	LHQNIVDVQYLYGVG	Class I	D Ciuffreda
				S9	# *			
1379-1387	IPFYGKAIPI	NS3	B51	A13	A*0101 B*0801 B*5101 C*0102 C*0701 #	IPFYGKAI &	B51	S Giugliano
				A2	B*4403 B*5101 C*1402 C*1601 #	IPFYGKAIP**L**	B51	D Yerly
1436-1447	GDVVVCATDALMTGF	NS3		A15	A*0101 B*5201 B*5701 C*0602 C*1202	ATDALMTGY	A1	G M Lauer, 2002
1442-1447	ATDALMTGY		A01	A15	A*0101 B*5201 B*5701 C*0602 C*1202 #	**V**ATDALMTGYTGDFD	Class I	A M Werthheimer
				C108	A*0101 A*2402 B*0801 B*3906 C*0701 C*0702 #	ATDALMTGF &	A1	S Giugliano
				C106	A*0101 A*0301 B*0702 B*0702 C*0702 C*0702 #	ATDALMTGF	A1	T Kuntzen
				C68	A*0101 A*0201 B*0801 B*5701 C*0602 C*0701 #	ATDALMTGY	A1	A L Cox
						ATDALMTGY	A1	E Barnes
						ATDALMTGY	A1	G M Lauer, 2004

Table S7: Overlap between genotype-3-specific CD8+ T-cell targets defined in this study and previously described HCV genotype-1-specific CD8+ targets.

HCV genotype-3-specific CD8+ epitopes and described genotype-1-specific CD8+ epitopes were classified by overlap (left coloured bar): 'overlapping' (blue), 'likely overlapping' (light blue, <20% sequence differences within targeted area), 'unlikely overlapping' (light red, >20% differences within targeted area, or less than 7 amino acids overlap), and 'not overlapping' (red). ***Left:*** For each epitope, viral region, position and genotype-3 sequence is given, as well as patients targeting the epitopes (colour coding: orange-SR, blue-acute, yellow chronic), and patient's HLA class-I types. ***Right:*** Published overlapping genotype-1 CD8+ epitopes are specified, including sequence, restricting HLA type and first author of the publication; overlap between epitopes is underlined and genotype-3/genotype-1 sequence differences marked bold. # CD8 restriction for marked patient experimentally defined; * Epitopes

previously described in (Humphreys et al. 2012); & Epitope previously described for genotype-1 and 3.
(M Battegay et al. 1995; Rehermann et al. 1996; K. M. Chang et al. 1997; Löhr et al. 1999; Grüner et al. 2000; Wertheimer et al. 2003; Sugimoto et al. 2003; Lauer et al. 2004; Cox et al. 2005; Kennedy et al. 2006; Kuntzen et al. 2007; Yerly et al. 2008; Ciuffreda et al. 2008; Giugliano et al. 2009; Neumann-Haefelin et al. 2008; Barnes et al. 2012; Guo et al. 2012)

GT3 CD4+ epitopes (Oxford Cohort)							GT1 epitopes described in corresponding regions		
Position	3a peptide sequence	Viral protein	Patient ID				Peptide (Literature)	HLA	First author
453-476	CPQRLSSCKPITFFRQGWGSLTDANITGPSD	E2	A15	DRB1*0701	DRB1*1502	#	no CD4 epitopes described		
			C106						
2603-2618	KRALYDVIQKLSIETM	NS5B	S11	DRB1*0404	DRB1*0701	# *	KL**PLAV**MGSSYGFQYSPGQR	Class-II	J Schulze zur Wiesch, 2007
							KL**PLAV**MGSSYGFQYSPGQR	Class-II	C L Day
66-90	PKARRSEGRSWAQPGYPWPLYGNEG	core	C5	DRB1*0101	DRB1*0701		RRQPIPKARR**P**EGR**T**WAQPG	Class-II	J Schulze zur Wiesch, 2012
			C12	DRB1*0301	DRB1*0401		RQPIPKVRR**P**EGR**T**	HLA-DR	J J Lasarte
			C37	DRB1*0401	DRB1*0407		K**V**RR**P**EGR**T**WAQPG	HLA-DR	J J Lasarte
			450			#	PEGR**T**WAQPGYPWPLYGNEG	Class-II	J Schulze zur Wiesch, 2007
			C68	DRB1*0101	DBR1*0701	# *	PEGR**T**WAQPGYPWPLYGNEGCGW	Class-II	H F Löhr
							PEGR**T**WAQPGYPWP	HLA-DR	J J Lasarte
							PEGR**T**WAQPGYPWPL	Class-II	P T F Kennedy
143-158	PVGGVARALAHGVRAL	core	A16				ADLMGYIPLVGAP**L**GG**A**ARA	Class-II	J Schulze zur Wiesch, 2007
			C6	DRB1*0801	DRB1*1101	# *	ADLMGYIPLVGAP**L**GG**A**AR	HLA-DR	F A Castelli
			C12	DRB1*0301	DRB1*0401	#	ADLMGYIPLVGAP**L**GG**A**ARA	Class-II	J Schulze zur Wiesch, 2012
			C13	DRB1*1504	DRB1*0701		LMGYIPLVGAP**L**GG**A**	Class-II	D Ciuffreda
			C18	DRB1*0301	DRB1*1602	#	LVGAP**L**GG**A**ARAL	Class-II	H F Löhr
			C19	DRB1*0403	DRB1*0701		LVGAP**L**GG**A**ARALAH		K Sugimoto
			C22	DRB1*0404	DRB1*1104	#	GAP**L**GG**A**ARALAHGVR**V**LED	Class-II	J Schulze zur Wiesch, 2007
			C23	DRB1*0701	DRB1*0801	#	GAP**L**GG**A**ARALAHGVR**V**LED	Class-II	A J MacDonald
			C27	DRB1*0101	DRB1*1501		GAP**L**GG**A**ARALAHGVR**V**LED	Class-II	C L Day
			331			# *	GAP**L**GG**A**ARALAHGVR**V**LED	Class-II	J Schulze zur Wiesch, 2012
			C70	DRB1*0101	DRB1*1501		GG**A**ARALAHGVR**V**LE	Class-II	D Ciuffreda
			C77	DRB1*0101		# *	ALAHGVR**V**L	Class-II	H F Löhr
							LAHGVR**V**LEDGVNYATGNLP	Class-II	J Schulze zur Wiesch, 2012
1423-1440	AYYRGLDVSVIPTAGDVV	NS3	C22	DRB1*0404	DRB1*1104		GINAVAYYRGLDVSVIPT**S**G	Class-II	J Schulze zur Wiesch, 2007
			S11	DRB1*0404	DRB1*0701	# *	GINAVAYYRGLDVSV	Class-II	A M Wertheimer
							GINAVAYYRGLDVSVIPT**S**G	Class-II	C L Day
							VAYYRGLDVSVIPT**S**	Class-II	A M Wertheimer
							LDVSVIPT**S**GDVVVVATDAL	Class-II	J Schulze zur Wiesch, 2007
							IPT**S**GDVVVVSTDALMTG	Class-II	N M Tabatabai
2548-2565	NQIRSVWEDLLEDTTTPI	NS5B	S2	DRB1*0701	DRB1*1454	# *	HARKAV**TH**I**N**SVWKDLLED**N**	Class-II	J Schulze zur Wiesch, 2012
							SVWKDLLED**NV**TPIDTTIMA	Class-II	C L Day
2893-2908	IIERLHGLSAFTLHSY	NS5B	S2	DRB1*0701	DRB1*1454	# *	PII**Q**RLHGLSAF**S**LHSYSPG	Class-II	J Schulze zur Wiesch, 2007
							PII**Q**RLHGLSAF**S**LHSYSPG	Class-II	J Schulze zur Wiesch, 2012

Table S8: Overlap between genotype-3-specific CD4+ T-cell targets defined in this study and previously described HCV genotype-1-specific CD4+ targets.
HCV genotype-3-specific CD4+ T-cell targets and described genotype-1-specific CD4+ targets and those without defined CD4/CD8 restricted were classified by overlap (left coloured bar): 'likely overlapping' (light blue, <20% sequence differences within targeted area), 'unlikely overlapping' (light pink, >20% differences within targeted area, or less than 7 amino acids overlap), and 'not overlapping' (pink). ***Left:*** For each epitope, viral region, position and genotype-3 sequence is given, as well as patients targeting the epitopes (colour coding: orange-SR, blue-acute, yellow chronic), and patient's HLA class-II types. ***Right:*** Published overlapping genotype-1 CD8+ epitopes are specified, including sequence, restricting HLA type and first author of the publication; overlap between epitopes is underlined and genotype-3/genotype-1 sequence differences marked bold. # CD4 restriction for marked patient experimentally defined; * Targets previously described in (Humphreys et al. 2012).
(Lasarte et al. 1998; Lamonaca et al. 1999; Löhr et al. 1999; Tabatabai et al. 1999; Day et al. 2002; MacDonald et al. 2002; Sugimoto et al. 2003; Wertheimer et al. 2003; Kennedy et al. 2006; Castelli et al. 2007; Schulze zur Wiesch et al. 2007; Ciuffreda et al. 2008; Schulze Zur Wiesch et al. 2012).

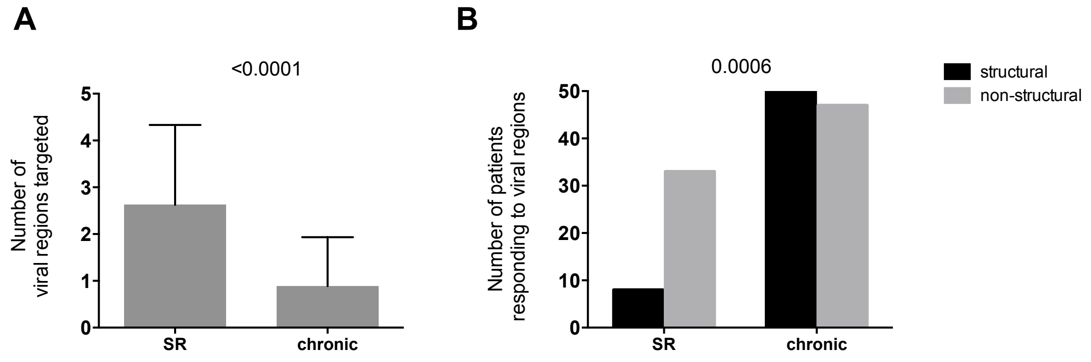

Figure S1: Comparison of breadth and targeted viral regions in spontaneously resolved individuals and patients with chronic HCV genotype-3 infection.
(A) Numbers of viral regions targeted in spontaneously resolved HCV infection and chronic HCV genotype-3 infection (unpaired t-test). (B) Comparison of numbers of patients targeting structural and non-structural regions in patient with chronic HCV genotype-3 infection and patients with spontaneously resolved HCV infection (Fisher's exact).

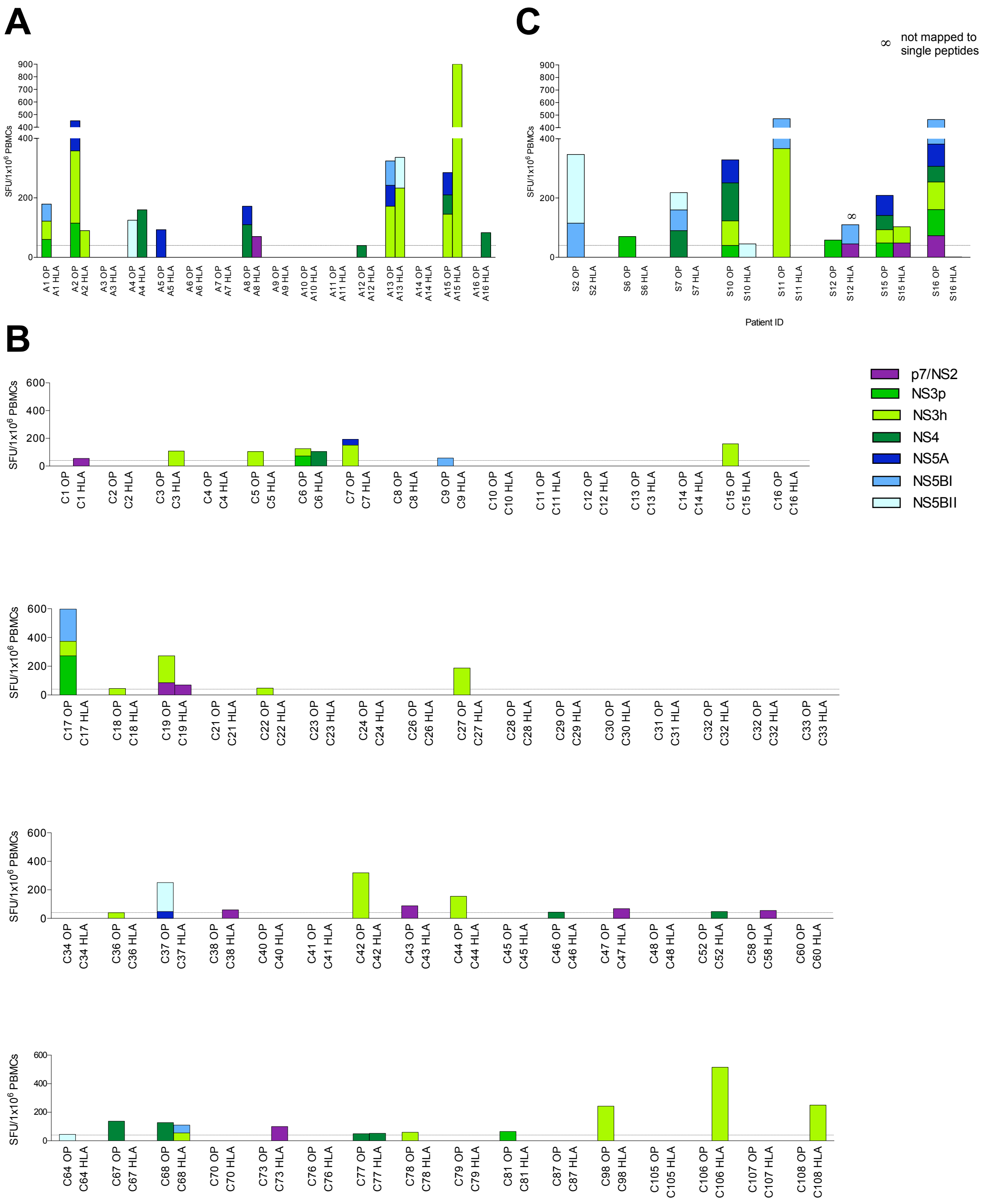

Figure S2: Comparison of responses to overlapping pools and HLA predicted peptides in acutely and chronically HCV genotype-3 infected patients, and patients with spontaneously resolved infection.

T-cell responses detected by IFNγ ELISpot assay to HLA predicted peptides and overlapping peptide pools (OP) were measured in patients with (A) acutely (n=16) and (B) chronically HCV genotype-3 infected (n=64) patients, and (C) spontaneously resolved infection (n=8). Responses to overlapping pools are compared to the HCV viral region in which the HLA predicted peptide falls (NS3p, NS3h, NS4, NS5a, NS5b1, NS5b2). SFU: Spot forming units.

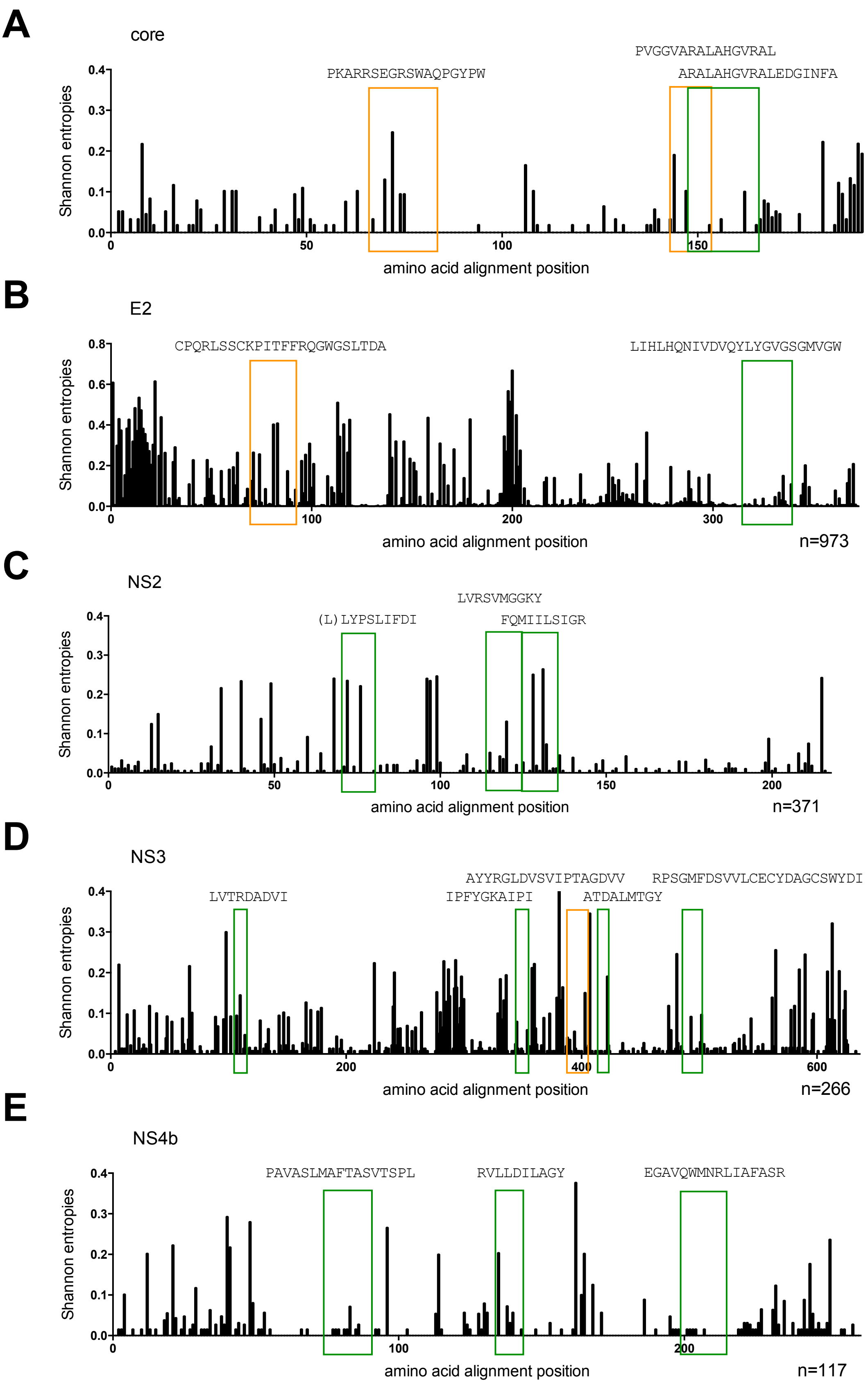
A
core
PVGGVARALAHGVRAL
PKARRSEGRSWAQPGYPW
ARALAHGVRALEDGINFA
Shannon entropies
0.4
0.3
0.2
0.1
0.0
0
50
100
150
amino acid alignment position
B
E2
CPQRLSSCKPITFFRQGWGSLTDA
LIHLHQNIVDVQYLYGVGSGMVGW
Shannon entropies
0.8
0.6
0.4
0.2
0.0
0
100
200
300
amino acid alignment position
n=973
C
NS2
LVRSVMGGKY
(L)LYPSLIFDI
FQMIILSIGR
Shannon entropies
0.4
0.3
0.2
0.1
0.0
0
50
100
150
200
amino acid alignment position
n=371
D
NS3
AYYRGLDVSVIPTAGDVV
RPSGMFDSVVLCECYDAGCSWYDI
LVTRDADVI
IPFYGKAIPI
ATDALMTGY
Shannon entropies
0.4
0.3
0.2
0.1
0.0
0
200
400
600
amino acid alignment position
n=266
E
NS4b
PAVASLMAFTASVTSPL
RVLLDILAGY
EGAVQWMNRLIAFASR
Shannon entropies
0.4
0.3
0.2
0.1
0.0
0
100
200
amino acid alignment position
n=117

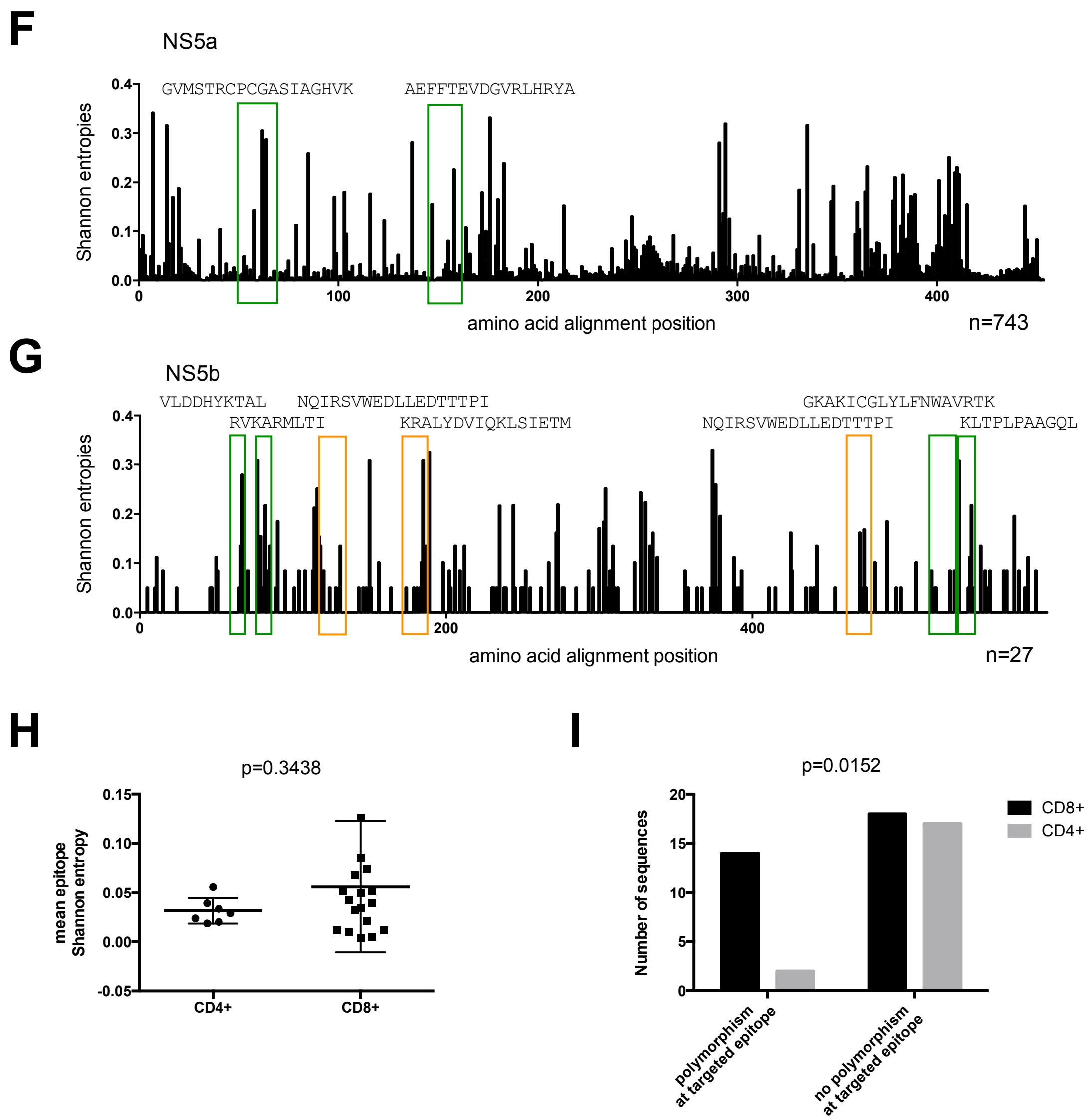

Figure S3: Evaluation of sequence heterogeneity at targeted HCV genotype-3-specific epitopes.

Sequence heterogeneity was evaluated by Shannon entropy scores in HCV viral regions (A) core, (B) E2, (C) NS2, (D) NS3, (E) NS4b, (F) NS5a and (G) NS5b on population level using Los Alamos genotype-3 sequences. The number of sequences used for each calculation is given. CD4+ (orange) and CD8+ (green) T-cell targets detected within the Oxford genotype-3 cohort are marked. (H) Mean Shannon entropy scores for CD4+ and CD8+ epitopes were calculated (p=0.3438, unpaired t-test). (I) Sequence polymorphisms within the Oxford cohort were evaluated at 16 CD8+ and 4 CD4+ T-cell targets in patients with detected T-cell responses. Sequence polymorphisms at CD4+ epitopes were detected in 2/19 sequenced patients, whereas polymorphisms at CD8+ epitopes were detected in 14/32 sequenced patients, respectively (p=0.0152, Fisher's exact).

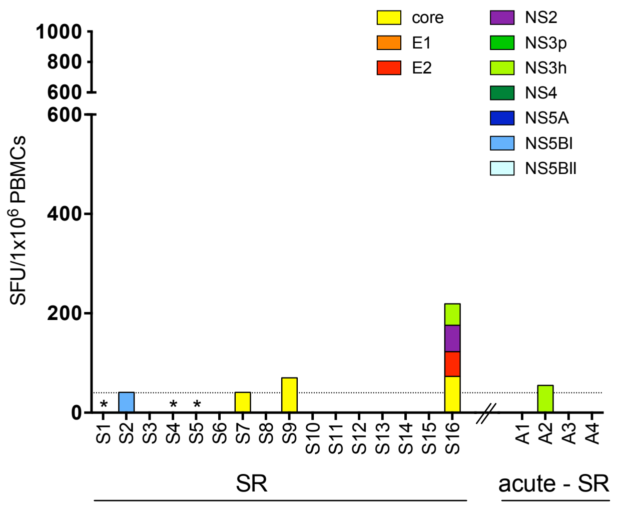

Figure S4: T-cell responses detected against HCV genotype-1 peptide sets in spontaneously resolved patients and acutely infected patients with subsequent resolution of infection.

Hepatitis C virus (HCV) genotype-1 specific T-cell responses were measured by IFNγ-ELISpot assays (spot-forming units (SFU)/10^6 peripheral blood mononuclear cells (PBMC)) using an HCV genotype-1b-specific peptide set spanning the entire HCV genome in 16 patients with spontaneously resolved infection (SR), and 4 patients acutely infected with HCV genotype-3 who subsequently spontaneously resolved infection (A-SR).

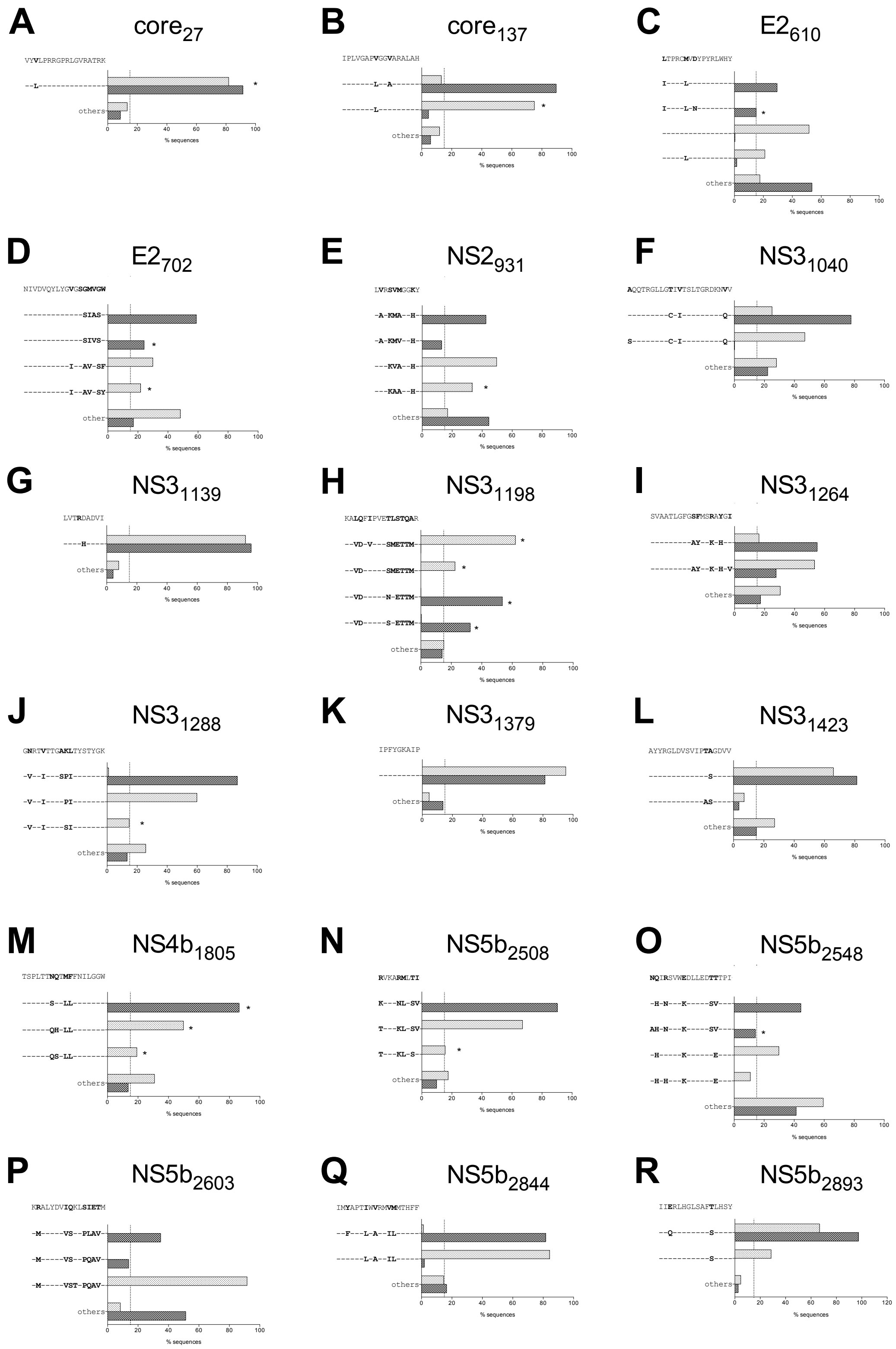

Figure S5: Hepatitis C virus (HCV) genotype-1 sequences variants at T-cell targets identified in spontaneously resolved patients.

HCV genotype-1 sequences were obtained from the Los Alamos database at T-cell targets identified in spontaneously resolved patients, to define common HCV genotype-1 sequence variants (frequencies >15%). For each T-cell target (A-R), HCV genotype-3 consensus sequence (top of each graph) and common HCV genotype-1 variants are specified. Where T-cell cross-reactivity to common variants has not been assessed experimentally due to lack of PBMC, sequences are marked with a star.

A

	Epitope sequence	Viral region	start AA	end AA	HLA type	number of publications	T cell targets in Oxford gt3 cohort	Restriction of HCV gt3 epitopes
CD8	YLLPRRGPRL	core	35	44	A2	10		
	GPRLGFRAT	core	41	49	B7	5		
	NEGLGWTGW	core	87	95	B44	5		
	ADLMGYIPLV	core	131	140	A2	11		
	LLALLSCLTV	core	178	187	A2	5		
	SMVGNWAKV	E1	363	371	A2	5		
	SLLAPGAKQNV	E2	401	411	A2	5		
	NTRPPLGNW	E2	541	549	B57	5		
	RDWAHNGL	NS2	957	964	B37	7		
	CINGVCWTV	NS3	1073	1081	A2	31		
	LLCPAGHAV	NS3	1169	1177	A2	5		
	HPNIEEVAL	NS3	1359	1367	B35	7		
	HSKKKCDEL	NS3	1395	1403	B8	8		
	KLVALGINAV	NS3	1406	1415	A2	32		
	ATDALMTGY	NS3	1436	1444	A1	7	+	CD8+
	LLFNILGGWV	NS4b	1807	1816	A2	7		
	VLSDFKTWL	NS4a	1987	1995	A2	7		
	ALYDVVTKL	NS5b	2594	2602	A2	11		
CD4+	NKRNTNRRPQDVKFPGGGQIVGGVYLLPRRGPRL	core	11	44	ND	9		
	KVRRPEGRTWAQPGYPWPLYGNEGLGWAGWLLSPRGS	core	67	103	ND	10	+	CD4+
	ADLMGYIPLVGAPLGGAARALAHGVRVLED	core	131	160	ND	12	+	CD4+
	ATRDGKLPATQLRRHIDLL	E1	247	265	ND	7		
	YFSMVGNWAKVLVVL	E1	361	375	ND	6		
	SSDLYLVTRHADVIP	NS3	1127	1141	ND	6		
	LETTMRSPVFTDNSSPPVVP	NS3	1201	1220	ND	6		
	PAAYAAQGYKVLVLNPSVAA	NS3	1241	1260	ND	8		
	LADAGCSGGAYDIIICDECHSTDAT	NS3	1300	1324	ND	8		
	EVIKGGRHLIFCHSKKKCD	NS3	1383	1401	ND	9		
	CNTCVTQTVDFSLDPTFT	NS3	1454	1471	ND	7		
	FVAPGERPSGMFD	NS3	1501	1513	ND	6		
	YELTPAETTVRLRAYMNTPGLPVAQD	NS3	1528	1553	ND	11		
	THIDAHFLSQTK	NS3	1566	1577	ND	6		
	ENLPYLVAYQATVCARAQAPPPSW	NS3	1581	1604	ND	10		
	PLLYRLGAVQNEITLTHP	NS3	1623	1640	ND	8		
	VIVGRVVLSGKPAIIPDREV	NS4a	1681	1700	ND	6		
	GLSTLPGNPAIASL	NS4a	1777	1790	ND	6		
	ALVVGVVCAAILRRHVGPGE	NS4a	1891	1910	ND	6		
	QCCDLDPQARVAIKSLTERL	NS5b	2661	2680	ND	6		

B

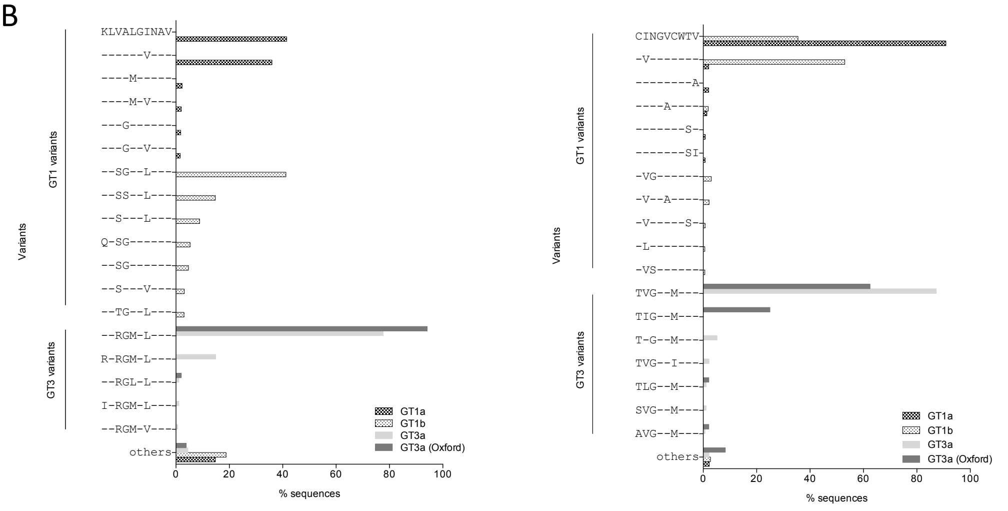

Figure S6: T-cell targets frequently detected in HCV genotype-1 infection are not targeted in HCV genotype-3 infection, likely due to sequence differences between genotypes.

(A) Comparison of CD8+ restricted epitopes dominant in HCV genotype-1 infection (defined as described in 5 or more publications on the IEDB). If the epitope was targeted in the Oxford HCV genotype-3 cohort, it is marked with +. Epitopes described as CD4+ restricted epitopes in the Oxford genotype-3 cohort falling into regions of CD8+ restricted epitopes described as HCV genotype-1 epitopes in the literature are marked in grey. (B) Comparison of circulating viral genotype-1 and genotype-3 sequences at dominant HCV genotype-1 epitopes. Sequences were obtained from the Los Alamos database, with additional in-house genotype-3 sequences.

Barnes, E. et al., 2012. Novel adenovirus-based vaccines induce broad and sustained T cell responses to HCV in man. *Science translational medicine*, 4(115), p.115ra1.

Battegay, M et al., 1995. Patients with chronic hepatitis C have circulating cytotoxic T cells which recognize hepatitis C virus-encoded peptides binding to HLA-A2.1 molecules. *Journal of virology*, 69(4), pp.2462–2470.

Castelli, F.A. et al., 2007. Differential capacity of T cell priming in naive donors of promiscuous CD4+ T cell epitopes of HCV NS3 and Core proteins. *European journal of immunology*, 37(6), pp.1513–1523.

Chang, K.M. et al., 1997. Immunological significance of cytotoxic T lymphocyte epitope variants in patients chronically infected by the hepatitis C virus. *The Journal of clinical investigation*, 100(9), pp.2376–2385.

Ciuffreda, D. et al., 2008. Polyfunctional HCV-specific T-cell responses are associated with effective control of HCV replication. *European journal of immunology*, 38(10), pp.2665–2677.

Cox, A.L. et al., 2005. Comprehensive analyses of CD8+ T cell responses during longitudinal study of acute human hepatitis C. *Hepatology*, 42(1), pp.104–112.

Day, C.L. et al., 2002. Broad Specificity of Virus-Specific CD4+ T-Helper-Cell Responses in Resolved Hepatitis C Virus Infection. *J. Virol.*, 76(24), pp.12584–12595.

Ge, D. et al., 2009. Genetic variation in IL28B predicts hepatitis C treatment-induced viral clearance. *Nature*, 461(7262), pp.399–401.

Giugliano, S. et al., 2009. Degree of cross-genotype reactivity of hepatitis C virus-specific CD8+ T cells directed against NS3. *Hepatology (Baltimore, Md.)*, 50(3), pp.707–716.

Grüner, N.H. et al., 2000. Association of hepatitis C virus-specific CD8+ T cells with viral clearance in acute hepatitis C. *The Journal of Infectious Diseases*, 181(5), pp.1528–1536.

Guo, Z. et al., 2012. DCs pulsed with novel HLA-A2-restricted CTL epitopes against hepatitis C virus induced a broadly reactive anti-HCV-specific T lymphocyte response. *PloS one*, 7(6), p.e38390.

Humphreys, I.S. et al., 2012. HCV genotype-3a T cell immunity: specificity, function and impact of therapy. *Gut*. Available at: http://www.ncbi.nlm.nih.gov/pubmed/22337948 [Accessed May 17, 2012].

Kennedy, P.T.F. et al., 2006. The influence of T cell cross-reactivity on HCV-peptide specific human T cell response. *Hepatology (Baltimore, Md.)*, 43(3), pp.602–611.

Kuntzen, T. et al., 2007. Viral Sequence Evolution in Acute Hepatitis C Virus Infection. *J. Virol.*, 81(21), pp.11658–11668.

Lamonaca, V. et al., 1999. Conserved hepatitis C virus sequences are highly immunogenic for CD4(+) T cells: implications for vaccine development. *Hepatology (Baltimore, Md.)*, 30(4), pp.1088–1098.

Lasarte, J.J. et al., 1998. Cellular immunity to hepatitis C virus core protein and the response to interferon in patients with chronic hepatitis C. *Hepatology (Baltimore, Md.)*, 28(3), pp.815–822.

Lauer, G.M. et al., 2004. High resolution analysis of cellular immune responses in resolved and persistent hepatitis C virus infection. *Gastroenterology*, 127(3), pp.924–936.

Löhr, H.F. et al., 1999. The viral clearance in interferon-treated chronic hepatitis C is associated with increased cytotoxic T cell frequencies. *Journal of hepatology*, 31(3), pp.407–415.

MacDonald, A.J. et al., 2002. CD4 T Helper Type 1 and Regulatory T Cells Induced against the Same Epitopes on the Core Protein in Hepatitis C Virus-Infected Persons. *Journal of Infectious Diseases*, 185(6), pp.720–727.

Neumann-Haefelin, C. et al., 2008. Analysis of the evolutionary forces in an immunodominant CD8 epitope in hepatitis C virus at a population level. *Journal of Virology*, 82(7), pp.3438–3451.

Rehermann, B. et al., 1996. Quantitative analysis of the peripheral blood cytotoxic T lymphocyte response in patients with chronic hepatitis C virus infection. *The Journal of clinical investigation*, 98(6), pp.1432–1440.

Schulze zur Wiesch, J. et al., 2007. Immunologic evidence for lack of heterologous protection following resolution of HCV in patients with non–genotype 1 infection. *Blood*, 110(5), pp.1559 –1569.

Schulze Zur Wiesch, J. et al., 2012. Broadly directed virus-specific CD4+ T cell responses are primed during acute hepatitis C infection, but rapidly disappear from human blood with viral persistence. *The Journal of experimental medicine*, 209(1), pp.61–75.

Sugimoto, K. et al., 2003. Suppression of HCV-specific T cells without differential hierarchy demonstrated ex vivo in persistent HCV infection. *Hepatology (Baltimore, Md.)*, 38(6), pp.1437–1448.

Tabatabai, N.M. et al., 1999. Functionally distinct T-cell epitopes within the hepatitis C virus non-structural 3 protein. *Human immunology*, 60(2), pp.105–115.

Wertheimer, A.M. et al., 2003. Novel CD4+ and CD8+ T-cell determinants within the NS3 protein in subjects with spontaneously resolved HCV infection. *Hepatology*, 37(3), pp.577–589.

Yerly, D. et al., 2008. Increased cytotoxic T-lymphocyte epitope variant cross-recognition and functional avidity are associated with hepatitis C virus clearance. *Journal of virology*, 82(6), pp.3147–3153.
